# A pan-cancer analysis of the expression of STAT family genes in tumors and their relationship to the tumor microenvironment

**DOI:** 10.3389/fonc.2022.925537

**Published:** 2022-09-13

**Authors:** Min Zhou, Ping Zhang, Mengting Da, Rui Yang, Yulian Ma, Jiuda Zhao, Tao Ma, Jiazeng Xia, Guoshuang Shen, Yu Chen, Daozhen Chen

**Affiliations:** ^1^ Department of Breast Surgery, The Affiliated Wuxi Maternity and Child Health Care Hospital of Nanjing Medical University, Wuxi, China; ^2^ Breast Disease Diagnosis and Treatment Center, Affiliated Hospital of Qinghai University and Affiliated Cancer Hospital of Qinghai University, Xining, China; ^3^ Research Institute for Reproductive Health and Genetic Diseases, The Affiliated Wuxi Maternity and Child Health Care Hospital of Nanjing Medical University, Wuxi, China; ^4^ Department of Obstetrics and Gynecology, Haidong No.2 People’s Hospital of Qinghai Province, Haidong, China; ^5^ Department of General Surgery and Translational Medicine Center, The Affiliated Wuxi No.2 People’s Hospital of Nanjing Medical University, Wuxi, China

**Keywords:** STAT, pan-cancer, prognosis, pathway analysis, tumor microenvironment

## Abstract

**Background:**

The signal transducer and activator of transcription (STAT) protein family, a group of seven members (STAT1, STAT2, STAT3, STAT4, STAT5A, STAT5B, and STAT6), has been widely used to investigate numerous biological functions including cell proliferation, differentiation, apoptosis, and immune regulation. However, not much is known about the role of the STAT family genes in pan-cancer.

**Methods:**

Tumor Immune Estimation Resource (TIMER), Sangerbox, cBioPortal, GSCALite, Xena Shiny, GeneMANIA, Gene Expression Profiling Interactive Analysis (GEPIA), and Metascape were used to analyze the relationship between STAT gene expression, clinical outcome, gene variation, methylation status, pathway activity, tumor immune infiltration, and microenvironment in different cancer types and screened drugs that could potentially influence STATs.

**Results:**

The Cancer Genome Atlas (TCGA) pan-cancer data showed that most STAT family genes were extensively changed in most tumors compared to the adjacent normal tissues. We also found that STAT gene expression could be used to predict patient survival in various cancers. The STAT gene family formed a network of interaction networks that was associated with several pathways. By mining the of Genomics Drug Sensitivity in Cancer (GDSC) database, we discovered a number of potential drugs that might target STAT regulators. Importantly, the close correlation between STATs and immunocell infiltration suggested the important role of dysregulation of STATs in tumor immune escape. Finally, the relation between STAT gene expression and the tumor microenvironment (TME) indicated that the higher expression of STAT regulators, the higher the degree of tumor stem cells.

**Conclusion:**

Considering these genomic alterations and clinical features of STAT family members across cancer types, it will be possible to change the relationship between STATs and tumorigenesis. It was beneficial to treat cancer by targeting these STAT regulators.

## Introduction

The signal transducer and activator of transcription (STAT) protein, which includes seven members (STAT1, STAT2, STAT3, STAT4, STAT5A, STAT5B, and STAT6), has been widely used to analyze different biological functions (1–3). Proteins contribute significantly to the pathogenesis of diseases, including cancer, autoimmune diseases, and infections, and their involvement in many signaling pathways downstream of cytokines, interleukin, and growth factors (4, 5). They function as cytokines, transcription factors, and regulating target genes, regulating the tumor suppressors or oncogenes.

In cancers, the activation of STAT genes is often observed and postulated that dysregulation of these factors may contribute to tumor progression at several levels (6, 7). STATs have different expression patterns and physiological functions. Numerous cancer cells, including breast, head and neck, and gastric, are abnormally high in STAT1 (8–10). A high expression of STAT1 is associated with improved clinical outcomes in most studies (11, 12). Despite that, results of other clinical trials with high STAT1 expression in cancer tissues are worse than those with low STAT1 expression in the cancerous tissues (13, 14). Over 70% of human cancers are estimated to have aberrant STAT3 activity (15). In many publications, the association between STAT3 and tumor growth and immune evasion has been clearly documented (16). Among the massive tumors reported, STAT3 dysregulation occurred in bladder, breast, cervix, head and neck, kidney, and stomach (17–22). The mechanism for STAT5 proteins is activated by numerous processes in human cancers, including alterations to epigenetic mechanisms, hormone-regulated transcription factors, proteolytic pathways, gene amplification, and aberrant expression of growth factors (23–25). Activated STAT5 causes oncogenic changes through transcriptional modifications and protein–protein interactions (PPIs). The STAT5 protein provides aberrant responses to DNA damage, invasion, metastasis, and epithelial-to-mesenchymal transition (EMT) (26). Recent studies have shown that STAT6 signaling reduces cancerous growth and/or metastasis in solid tumors of the gastrointestinal tract, the breast, the lung, and the prostate, suggesting that STAT6 signaling could prevent these cancers (27–30). Historically, there are limited numbers of reports relating STAT2 and STAT4 dysregulation with the clinical characteristics and prognosis of human cancer. The role of STAT family genes and their mechanisms of action are still not fully explained. The family genes for STAT were previously studied but only for the individual cancer type and did not include multitypes of cancer compared.

**Figure 1:**
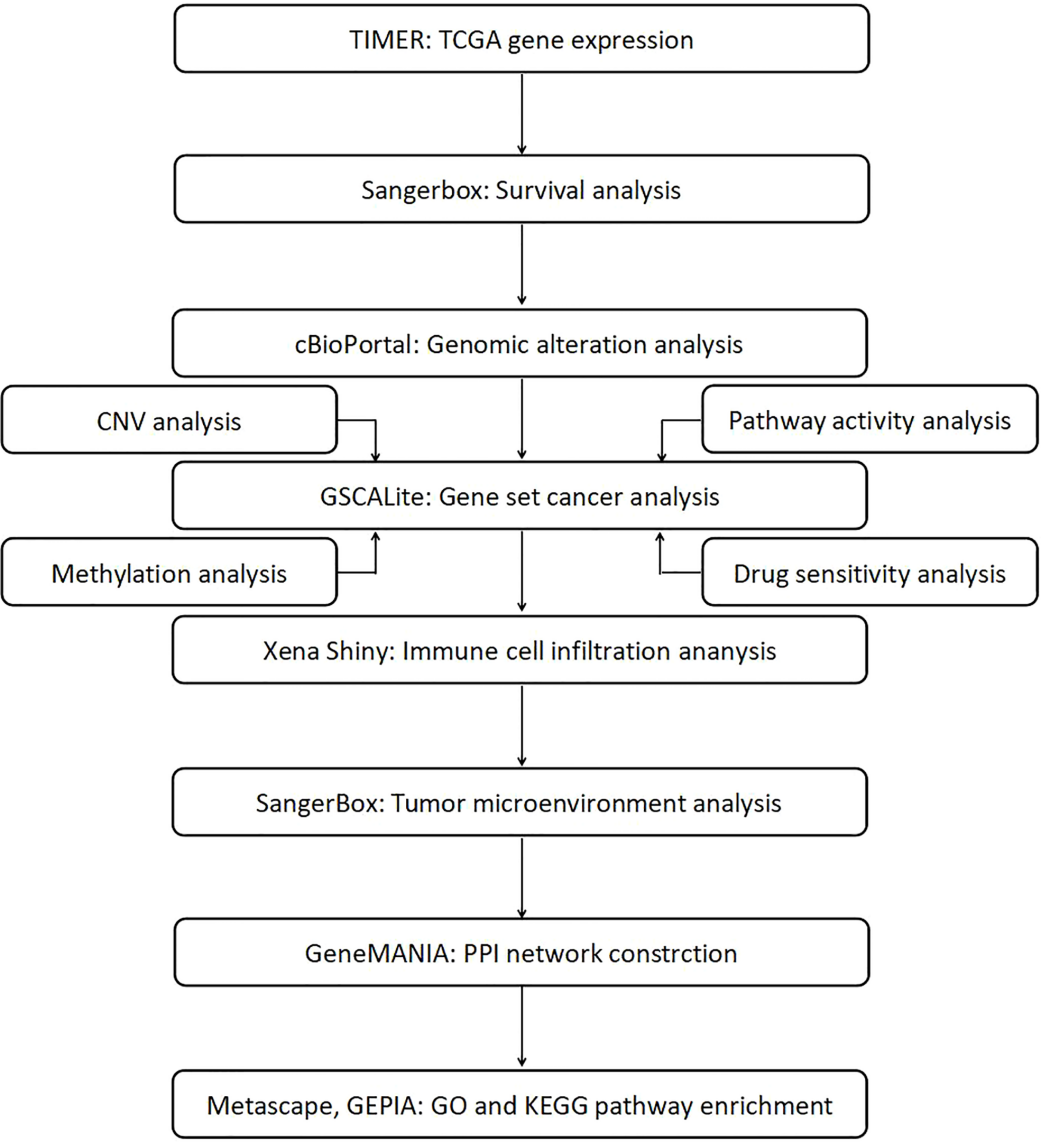
Workflow chart of the study.

In this pan-cancer study, we attempted to establish a role of STATs in different cancer types and determine the cellular mechanisms and functions of each STAT family gene and its interacting molecules in carcinogenesis. Our study examined the interrelationships between STAT expression, clinical outcome, gene variation, methylation status, pathway activity, immune infiltration, microenvironment of different cancers, and the potential impact of drugs on STATs. The workflow chart of this study was summarized in **Figure 1**.

## Methods

### Gene expression and survival analysis

The TIMER database (https://cistrome.shinyapps.io/timer/) includes 10,897 samples across 32 cancer types from The Cancer Genome Atlas (TCGA) to estimate the STAT family gene expression patterns (31, 32). We analyzed the differential expression of any gene of interest in tumors and adjacent normal tissues using the Diff Exp module provided by TCGA. In box plots, gene expression levels were captured, in Wilcoxon tests being used to determine if there were significant differences between levels. Based on the expression status of the STATs family, we performed a survival analysis using the Sangerbox database (http://vip.sangerbox.com/home.html). A median gene RNA-seq by Expectation Maximization (RSEM) value was used to classify tumor samples into high and low groups. The overall survival (OS) outcomes were analyzed by calculating the Hazard Ratio (HR) and 95% CI, along with a log-rank p-value.

### Genomic alteration analysis

cBioPortal (http://www.cbioportal.org/) was used to analyze genomic changes to STAT genes in pan-cancer (33). All types of Pan-Cancer Atlas available (32 studies and 10,967 samples) were selected for calculation. According to standard software parameters, altered frequencies of each gene were determined and the fraction of genome altered and survival curves were calculated.

### Gene set cancer analysis

By using GSCALite (http://bioinfo.life.hust.edu.cn/web/GSCALite/), the Genotype-Tissue Expression (GTEx) database was analyzed for expression levels of STAT family genes in each tissue (34). The STAT family genes were investigated for single-nucleotide variations (SNVs), copy number variations (CNVs), methylation, pathway function, and drug sensitivity. In selected cancer types, the SNV module displays the frequency and variant types of each gene set. The statistics of heterozygous and homozygous CNV of each cancer type were displayed as pie charts for gene sets on the CNV module. For selected cancer types, the Methylation module examined differences in methylation between tumors and matching normal tissue, the relationship between methylation and expression, and the impact of methylation on OS. The Pathway Activity module displayed differences in gene expression across pathway activity categories (activation and inhibition), which were determined by pathway scores. Drug sensitivity and gene expression profiling data from cancer cell lines in GDSC were combined in the Drug Sensitivity module. Spearman’s correlation analysis was used to compare the expression of each gene in the gene set to the sensitivity to small molecules and drugs [50% inhibiting concentration (IC50)].

### Immune cell infiltration

With Xena Shiny (https://shiny.hiplot.com.cn/ucsc-xena-shiny/), we could analyze immune cell infiltration across 32 cancer types from TCGA. We used the module TCGA: Associations between Molecular Profile and Immune Signature module of Xena Shiny to evaluate the interrelationships between STAT expression in 20 immune cells and their infiltration by using “CIBERSORT.” Pearson’s correlation coefficient of gene and immune infiltration score in each tumor was also generated.

### Tumor microenvironment analysis

The stromal and immune scores are positively related to the stromal and immune components in the tumor microenvironment (TME). In light of estimate scores, a composite score combining stromal scores and immune scores can determine the relative proportion of stromal and immune components in the TME. Sangerbox tool was used to calculate stromal, immune, and estimate scores. Spearman’s correlation coefficient of gene and immune infiltration score in each tumor was also generated by the Sangerbox tool. The Spearman correlation test was used to determine the association between tumor stemness and each STAT expression. An RNA stemness score (RNAss) based on mRNA expression and a DNA stemness score (DNAss) based on DNA methylation patterns were used in this pan-cancer study to measure tumor stemness.

### GeneMANIA analysis

GeneMANIA was used in bioinformatics methods to predict functions for genes or gene lists, as well as to construct PPI networks (35).

### Gene Ontology (GO) and Kyoto Encyclopedia of Genes and Genomes (KEGG) pathway enrichment

To perform enrichment analyses, related genes of STAT families were discovered using GEPIA (http://gepia.cancer-pku.cn/index.html) (36). Metascape (http://metascape.org) was used to analyze the function of STAT members and related genes (37). Gene Ontology (GO) analysis in Metascape could identify STATs and the similar genes by three categories, including molecular function (MF), cellular component (CC), and biological process (BP). By analyzing the Kyoto Encyclopedia of Genes and Genomes (KEGG), we identified the signaling pathways associated with STAT factors, as well as related genes.

### Statistical analysis

The Spearman’s correlation test or the Pearson’s correlation test was used to analyze correlations. Cox proportional hazards models were calculated to determine survivorship risk and HR. Each variable was analyzed using survival plots and compared with log-rank tests. Once two sets of data were detected, a p-value of 0.05 was declared significant.

## Results

### Expression of STAT genes that were extensively changed in pan-cancer

**Figure 2:**
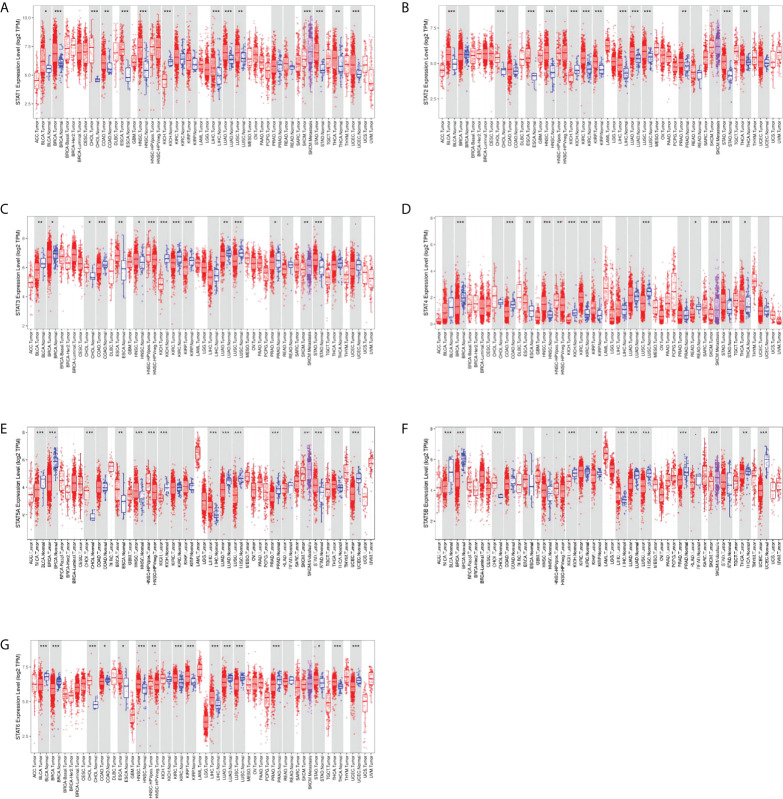
Differential expression of STAT genes in human normal organ tissues. **(A)** STAT1; **(B)** STAT2; **(C)** STAT3; **(D)** STAT4; **(E)** STAT5A; **(F)** STAT5B; **(G)** STAT6. *p < 0.05, **p < 0.01, ***p < 0.001.

We examined the expression levels of STAT family genes in all 33 cancer types available in TCGA pan-cancer data. Compared to other adjacent normal tissues, STATs tended to be extensively changed, suggesting a statistically significant difference in nearly half of all pan-cancers. STAT1 was highly expressed in bladder urothelial carcinoma (BLCA), breast invasive carcinoma (BRCA), cholangiocarcinoma (CHOL), colon adenocarcinoma (COAD), esophageal adenocarcinoma (ESCA), head and neck squamous cell carcinoma (HNSC), liver hepatocellular carcinoma (LIHC), lung adenocarcinoma (LUAD), lung squamous cell carcinoma (LUSC), stomach adenocarcinoma (STAD), thyroid carcinoma (THCA), and uterine corpus endometrial carcinoma (UCEC) and poorly expressed in kidney chromophobe (KICH), and the difference was statistically significant (**Figure 2A**). STAT2 was significantly upregulated in BLCA, CHOL, ESCA, HNSC, kidney renal clear cell carcinoma (KIRC), kidney renal papillary cell carcinoma (KIRP), LIHC, LUAD, LUSC, prostate adenocarcinoma (PRAD), and THCA but significantly downregulated in CHOL (**Figure 2B**). STAT3 expression remained high in CHOL, ESCA, HNSC, and STAD and low in BLCA, BRCA, COAD, KICH, KIRC, KIRP, LUAD, LUSC, and PRAD, and the difference was statistically significant (**Figure 2C**). STAT4 was significantly upregulated in ESCA, HNSC, KIRC, KIRP, STAD, and THCA but significantly downregulated in BRCA, COAD, KICH, LUSC, and rectum adenocarcinoma (READ) (**Figure 2D**). STAT5A was highly expressed in CHOL, ESCA, HNSC, LIHC, STAD, and THCA and poorly expressed in BLCA, BRCA, KICH, LUAD, LUSC, PRAD, and UCEC, and the difference was statistically significant (**Figure 2E**). STAT5B expression remained high in CHOL and LIHC and low in BLCA, BRCA, KICH, KIRP, LUAD, LUSC, PRAD, and THCA, and the difference was statistically significant (**Figure 2F**). STAT6 was significantly upregulated in CHOL, ESCA, HNSC, KIRC, KIRP, LIHC, STAD, and THCA but significantly downregulated in BLCA, BRCA, COAD, LUAD, LUSC, PRAD, and UCEC (**Figure 2G**).

### Prognostic potential of STAT family genes in pan-cancer

**Figure 3:**
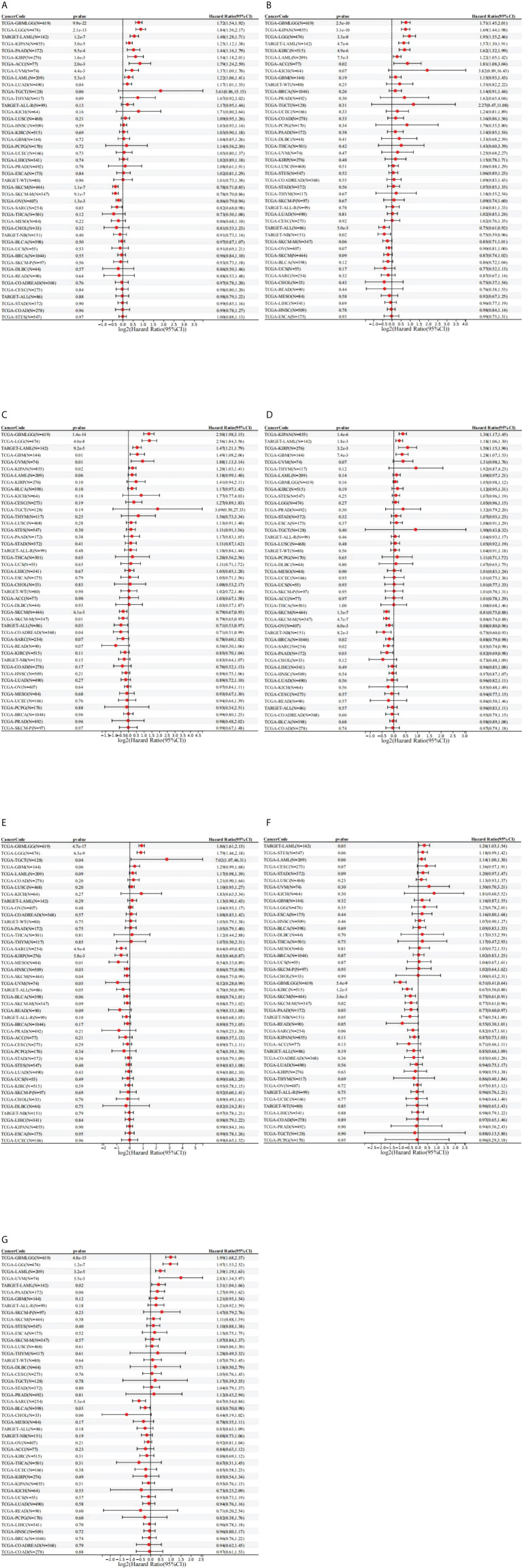
Prognostic role of STAT expression in pan-cancer. **(A)** STAT1; **(B)** STAT2; **(C)** STAT3; **(D)** STAT4; **(E)** STAT5A; **(F)** STAT5B; **(G)** STAT6.

To investigate the prognostic value of the seven STAT factors in pan-cancer, survival analysis was performed. High expression of STAT1 was significantly linked with the shortened OS in glioma (GBMLGG) [HR = 1.72 (1.54, 1.92), p = 9.9e-22], brain lower-grade glioma (LGG) [HR = 1.84 (1.56, 2.17), p = 2.1e-13], LUAD [HR = 1.17 (1.01, 1.35), p = 0.04], KIRP [HR = 1.54 (1.18, 2.01), p = 1.6e-3], pan-kidney cohort (KICH+KIRC+KIRP) (KIPAN) [HR = 1.25 (1.12, 1.38), p = 5.0e-5], uveal melanoma (UVM) [HR = 1.37 (1.09, 1.70), p = 4.4e-3], acute myeloid leukemia (LAML) [HR = 1.22 (1.06, 1.41), p = 5.3e-3], and adrenocortical carcinoma (ACC) [HR = 1.79 (1.24, 2.59), p = 2.0e-3], while lower STAT1 expression was significantly associated with lower OS rates in sarcoma (SARC) [HR = 0.82 (0.68, 0.98), p = 0.03], skin cutaneous melanoma (SKCM) [HR = 0.78 (0.71, 0.85), p = 1.1e-7], and ovarian serous cystadenocarcinoma (OV) [HR = 0.86 (0.79, 0.94), p = 1.3e-3] (**Figure 3A**). Higher STAT2 expression was significantly associated with poorer OS in GBMLGG [HR = 1.71 (1.45, 2.01), p = 2.5e-10], LGG [HR = 1.95 (1.55, 2.46), p = 3.3e-8], KIPAN [HR = 1.69 (1.44, 1.98), p = 3.1e-10], KIRC [HR = 1.62 (1.32, 1.99), p = 4.9e-6], LAML [HR = 1.22 (1.05, 1.42), p = 7.5e-3], and ACC [HR = 1.81 (1.08, 3.04), p = 0.02] (**Figure 3B**). GBMLGG, LGG, KIPAN, glioblastoma multiforme (GBM), and UVM patients with high STAT3 expression had worse OS [HR = 2.50 (1.98, 3.15), p = 1.4e-14; HR = 2.56 (1.84, 3.56), p = 4.0e-8; HR = 1.20 (1.03, 1.41), p = 0.02; HR = 1.49 (1.08, 2.06), p = 0.01; HR = 1.88 (1.13, 3.14), p = 0.01, respectively] than those with low STAT3 expression, while colon adenocarcinoma/rectum adenocarcinoma esophageal carcinoma (COADREAD) and SKCM patients with low STAT3 expression had worse OS [HR = 0.71 (0.51, 0.99), p = 0.04; HR = 0.79 (0.67, 0.93), p = 6.1e-3), respectively] than those with high STAT3 expression (**Figure 3C**). High expression of STAT4 was significantly linked with the shortened OS in KIRP [HR = 1.50 (1.15, 1.96), p = 3.2e-3], KIPAN [HR = 1.30 (1.17, 1.45), p = 1.4e-6], and GBM [HR = 1.28 (1.07, 1.53), p = 7.4e-3], while lower STAT4 expression was significantly associated with lower OS rates in BRCA [HR = 0.88 (0.79, 0.98), p = 0.02], SARC [HR = 0.85 (0.74, 0.98), p = 0.02], SKCM [HR = 0.81 (0.75, 0.88), p = 1.3e-7], OV [HR = 0.88 (0.80, 0.96), p = 6.0e-3], and pancreatic adenocarcinoma (PAAD) [HR = 0.82 (0.69, 0.98), p = 0.03] (**Figure 3D**). Higher STAT5A expression was significantly associated with poorer OS in GBMLGG [HR = 1.86 (1.61, 2.15), p = 4.7e-17], LGG [HR = 1.79 (1.46, 2.18), p = 6.3e-9], and testicular germ cell tumors (TGCTs) [HR = 7.02 (1.07, 46.31), p = 0.04], while low expression of STAT5A was significantly associated with lower OS rates in SARC [HR = 0.64 (0.49, 0.82), p = 4.9e-4], KIRP [HR = 0.63 (0.46, 0.87), p = 5.8e-3], HNSC [HR = 0.86 (0.75, 0.98), p = 0.03], SKCM [HR = 0.86 (0.75, 0.99), p = 0.04], mesothelioma (MESO) [HR = 0.54 (0.33, 0.89), p = 0.01], and UVM [HR = 0.52 (0.28, 0.99), p = 0.05] (**Figure 3E**). GBMLGG, KIRC, SKCM, and PAAD patients with low STAT5B expression had worse OS [HR = 0.51 (0.41,0.64), p = 5.4e-9; HR = 0.67 (0.56, 0.80), p = 1.2e-5; HR = 0.75 (0.61, 0.91), p = 3.6e-3; HR = 0.77 (0.60, 0.97), p = 0.03, respectively] than those with high STAT5B expression (**Figure 3F**). High expression of STAT6 was significantly linked with the shortened OS in GBMLGG [HR = 1.99 (1.68, 2.37), p = 4.8e-15], LGG [HR = 1.97 (1.53, 2.52), p = 1.2e-7], UVM [HR = 2.83 (1.34, 5.97), p = 5.5e-3], and LAML [HR = 1.39 (1.19, 1.63), p = 3.2e-5], while lower STAT6 expression was significantly associated with lower OS rates in SARC [HR = 0.67 (0.54, 0.84), p = 5.3e-4] and BLCA [HR = 0.83 (0.70, 0.98), p = 0.03) (**Figure 3G**).

### Genetic alteration analysis of the STATs in pan-cancer

**Figure 4:**
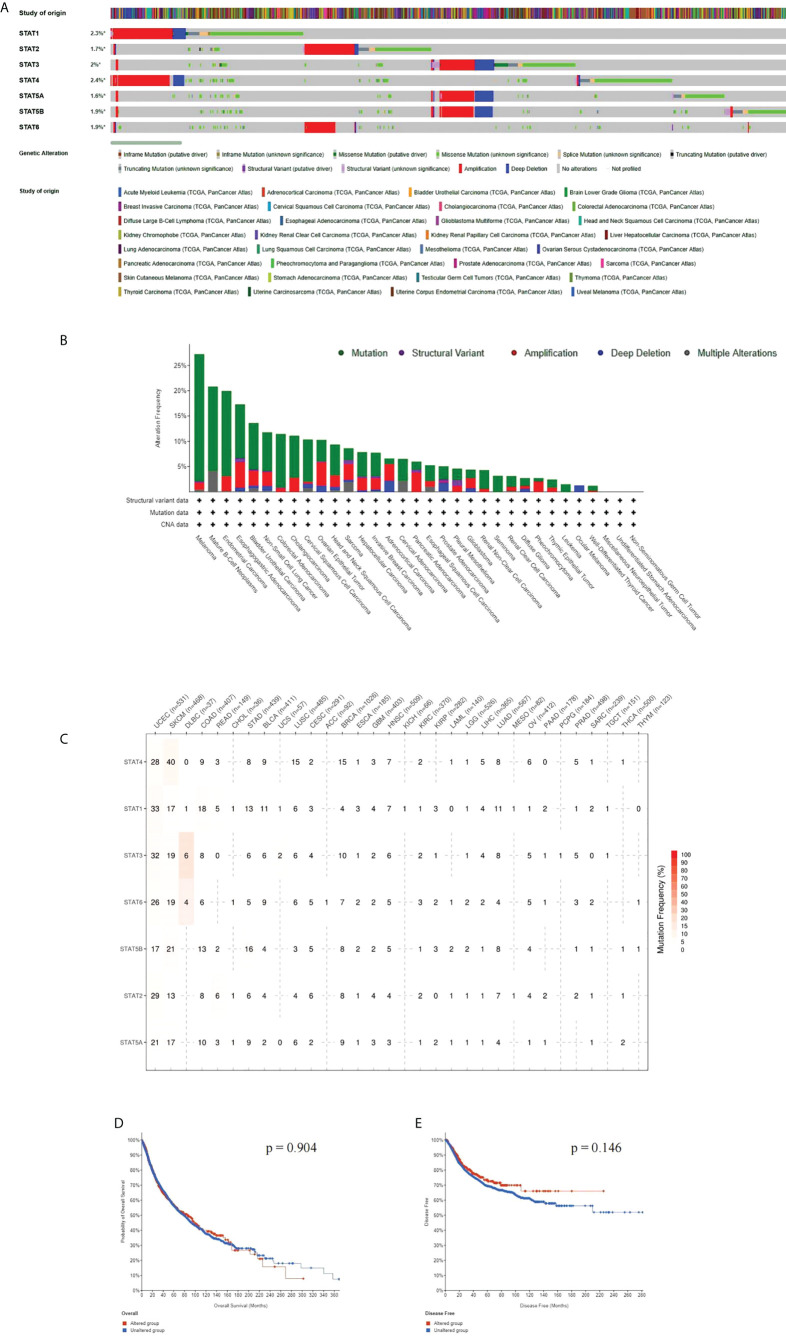
Alterations of STAT family genes. **(A)** STAT genetic alterations across TCGA cancer studies. **(B)** The alteration frequency of STAT genes plotted in pan-cancer with different cancer types. **(C)** SNV oncoplot using GSCALite website. **(D, E)** Genetic alterations in STATs were not associated with overall survival (OS) and disease-free survival (DFS).

Using the cBioPortal tool on 10,967 samples in 32 studies, we calculated the mutation frequency of the seven STAT genes. The gene of STAT genes was altered in 1,003 (9%) samples; STAT1, STAT2, STAT3, STAT4, STAT5A, STAT5B, andSTAT6 were altered in 2.3%, 1.7%, 2.0%, 2.4%, 1.6%, 1.9%, and 1.9% of the demanded pan-cancer samples (**Figure 4A**). We found that mutations of STAT family genes more frequently occur in melanoma, mature B-cell neoplasms, UCEC, esophagogastric adenocarcinoma, BLCA, NSCLC, COADREAD, CHOL, and cervical squamous cell carcinoma (>10%) (**Figure 4B**). An analysis of SNVs found the highest mutation levels in STATs in tumor tissues in UCEC and SKCM. These mutations increased expression levels in genes. In the greatest number of tumors, STAT4 was correlated with tumor growth, then STAT1, STAT3, STAT6, STAT5B, STAT2, and STAT5A (**Figure 4C**). The alterations were not related to the survival of STAT family gene mutations, and the OS and disease-free survival (DFS) of patients with alterations were not shortened as opposed to those without alterations (all p > 0.05; **Figures 4D, E** **)**.

### CNV of STAT genes

**Figure 5:**
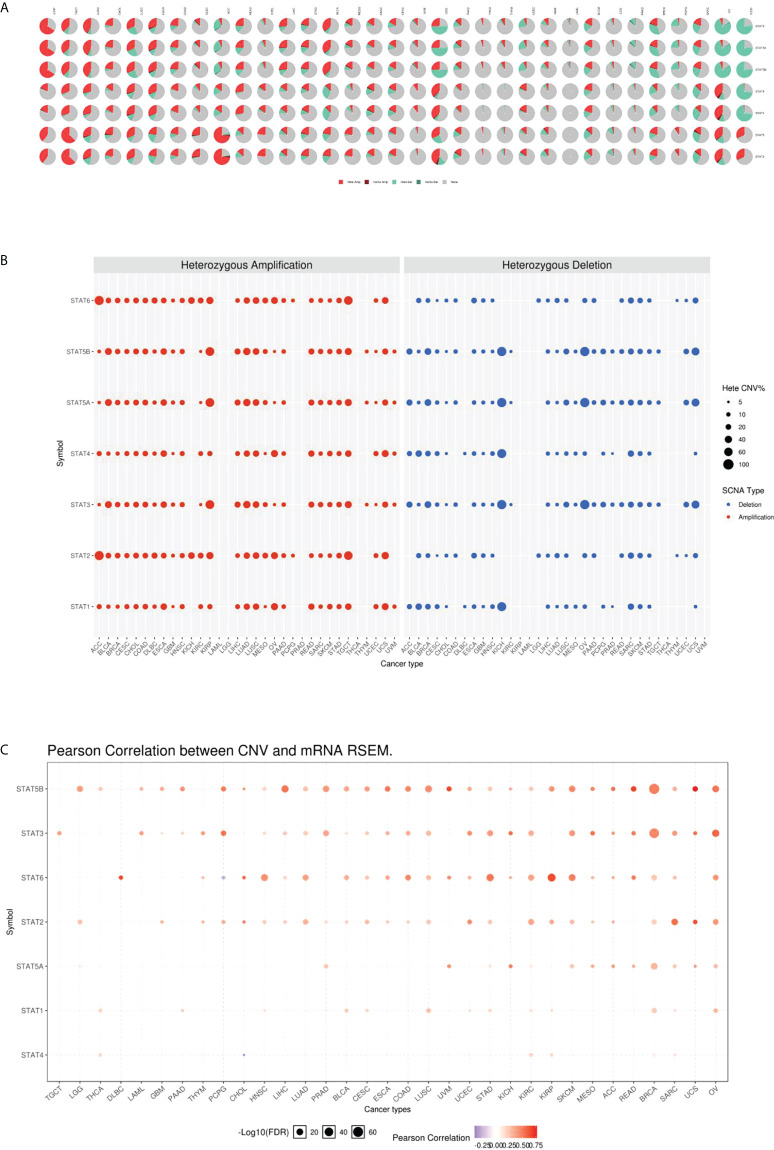
CNV underlies the dysregulation of STAT members. **(A)** CNV distribution in pan-cancer. **(B)** The percentage of amplification and deletion of heterozygous CNVs for each STAT gene in each cancer. **(C)** CNV correlation with mRNA expression.

According to the CNV pie chart, most types of CNV were heterozygous amplifications and deletions (**Figure 5A**). ACC and TGCT showed heterozygous amplification of STAT6; KIRP displayed STAT5A, STAT5B, and STAT3; and ACC and TGCT showed greater than 50% amplification of STAT2 (p < 0.05, **Figure 5B**). OV and KICH exhibited heterozygous deletions of STAT5B, STAT5A, and STAT3; STAT4 and STAT1 in KICH were both greater than 50% deleted (p < 0.05, **Figure 4B**). As a result of CNV, expression levels of every STAT factor increased. This interrelationship has been found in the largest number of tumors for STAT5B, followed by STAT3, STAT6, STAT2, STAT5A, STAT1, and STAT4 (**Figure 5C**).

### Methylation analysis of STAT regulators

**Figure 6:**
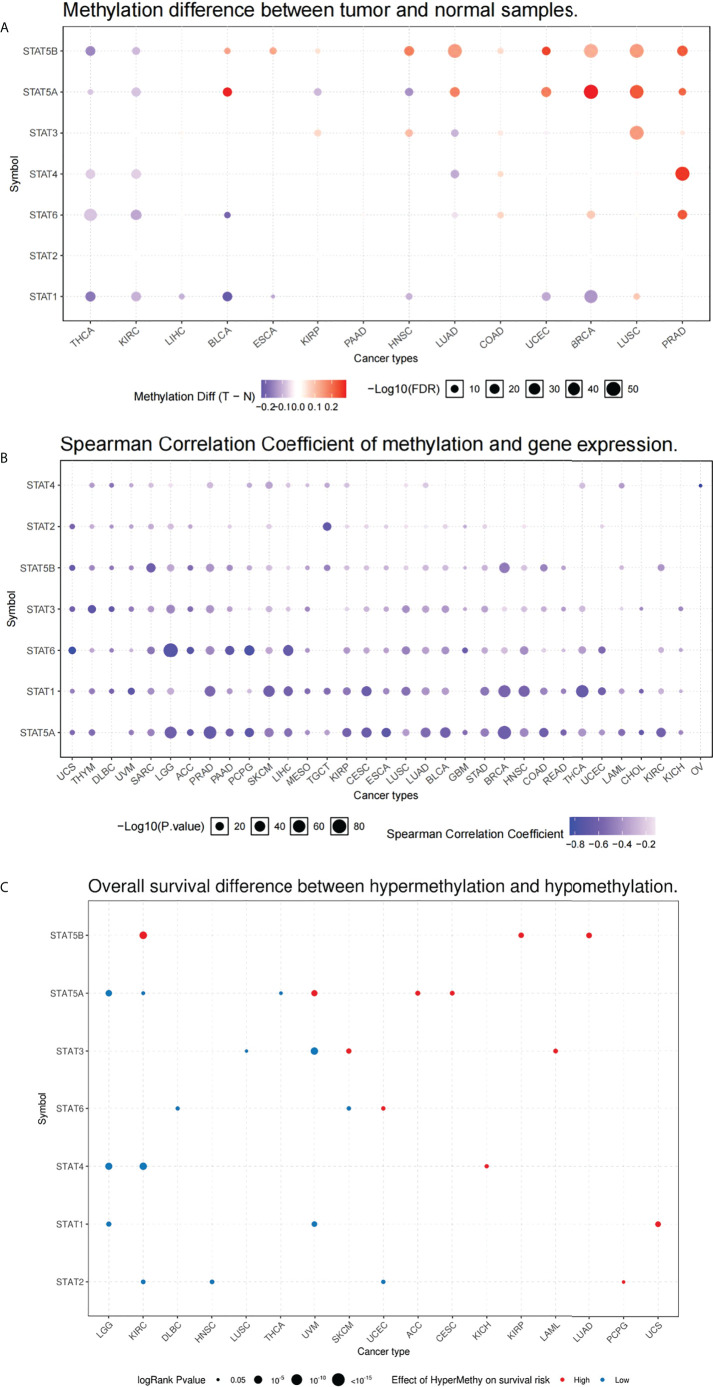
Methylation of melatonergic regulators. **(A)** Methylation difference between tumor and normal samples. **(B)** Spearman’s correlation coefficient of methylation and STAT gene expression. **(C)** Overall survival difference between hypermethylation and hypomethylation.

To identify the epigenetic regulation in TCGA database, the methylation characteristics of STAT genes were examined. In different tumors, methylation of STAT genes was heterogeneous: in PRAD, LUSC, BRCA, UCEC, and COAD, more highly hypermethylated genes could be found, while in THCA, KIRC, and LIHC, more highly hypomethylated genes can be found (**Figure 6A**). A correlation analysis of the methylation levels and mRNA expression levels revealed that a large proportion of genes, especially STAT1, STAT5A, and STAT6, correlated negatively with the methylation levels (**Figure 6B**). A survival study of various types of tumors showed that hypermethylation of STAT5A and STAT5B was mainly linked to poorer survival, while hypomethylation of STAT1, STAT2, and STAT4 was generally linked to shorter survival (p < 0.05, **Figure 6C**).

### Pathway activity analysis

**Figure 7:**
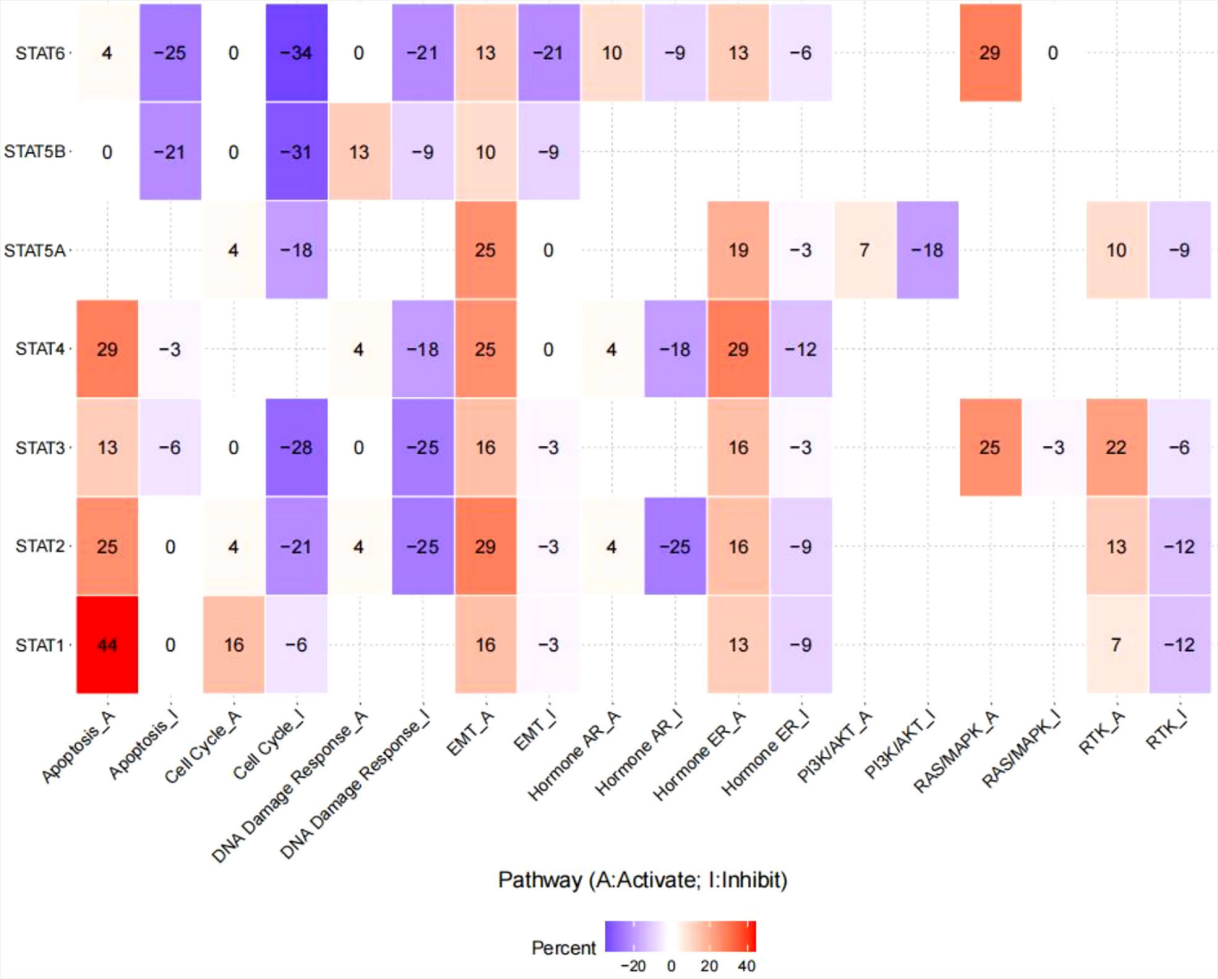
Pathway network between STAT family regulators.

The linked pathway network indicated that STAT family genes acted significantly in nine famous cancer-related signaling pathways. For STAT1, the principal inactivated pathways were apoptosis, cell cycle, and EMT. STAT2 was mostly involved in the activation of apoptosis and EMT, while the key pathways inactivated were cell cycle, DNA damage response, and hormone Androgen Receptor (AR). STAT3 was mostly involved in the activation of EMT, hormone Estrogen Receptor (ER), rat sarcoma/mitogen activated protien kinase RAS/MARK, and Receptor tyrosine kinase (RTK), while the major inactivated pathways were cell cycle and DNA damage response. STAT4 was generally involved in the inhibition of DNA damage response and hormone AR, while the main activated pathways were apoptosis, EMT, and hormone ER. STAT5A was mainly involved in the activation of EMT and hormone ER, while the main inactivated pathways were cell cycle and PI3K/AKT. In STAT5B, the primary inactivated pathways were apoptosis and cell cycle. STAT6 was mostly involved in RAS/MARK activation, while the main inactivated pathways were apoptosis, cell cycle, DNA damage response, and EMT (all p < 0.05, **Figure 7**).

### Drug sensitivity analysis

**Figure 8:**
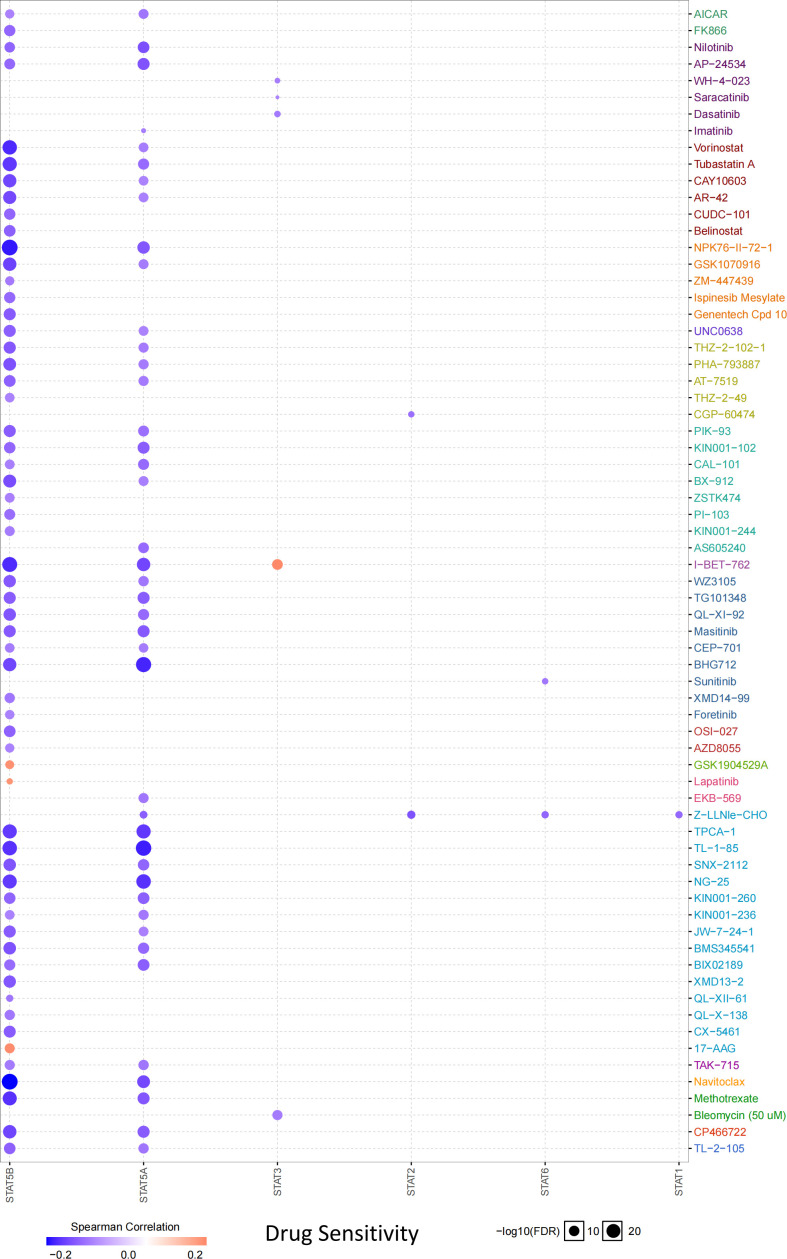
Drug sensitivity analysis of STAT family regulators from GDSC IC50 drug data.

According to the drug sensitivity analysis, low levels of STAT5B and STAT5A showed resistance to 56 and 42 drugs, respectively. Drug resistance toward vorinostat, tubastatin A, NPK76-II-72-1, I-BET-762, TPCA-1, TL-1-85, NG-25, navitoclax, and methotrexate negatively correlated with STAT5B expression (correlation coefficient >-0.20). Similarly, drug resistance toward BHG712, TPCA-1, TL-1-85, and NG-25 negatively related to the STAT5A expression (correlation coefficient >-0.20) (**Figure 8**).

### Correlation between the expression levels of STATs and immune cell infiltration in pan-cancer

**Figure 9:**
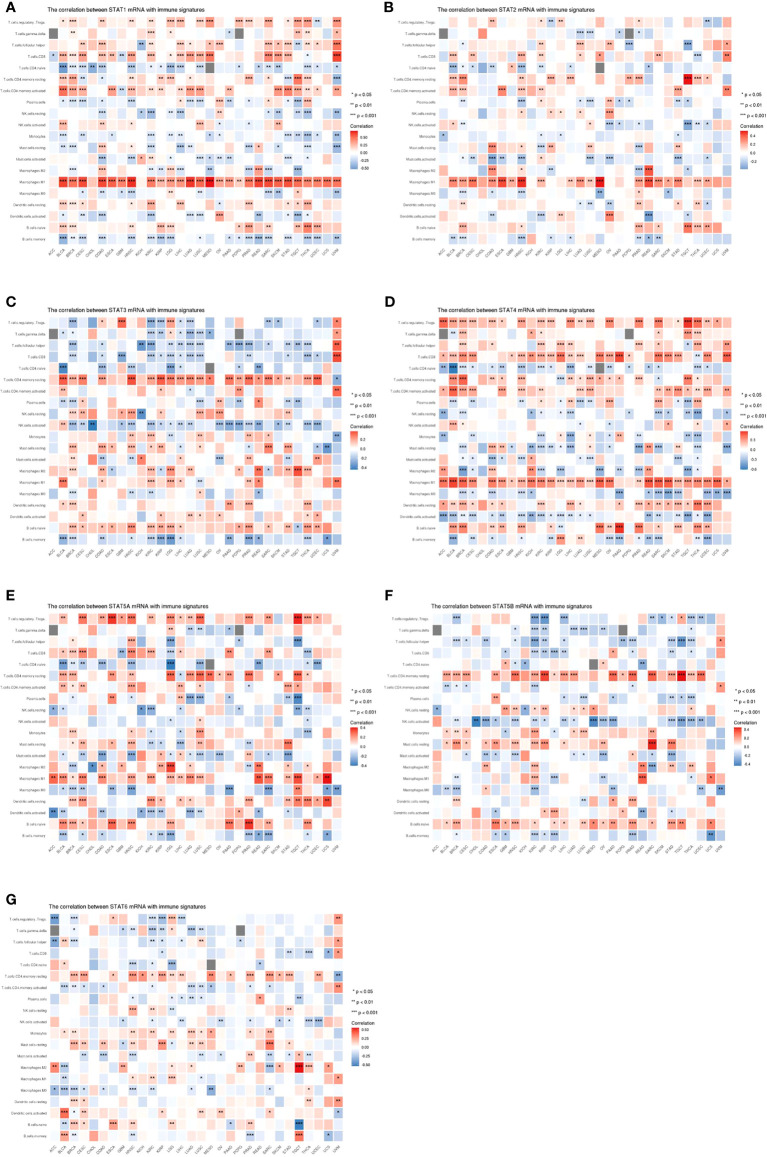
Correlation between the expression levels of signal transducer and activator of transcription (STAT) genes and immune infifiltrationin pan-cancer. **(A)** STAT1; **(B)** STAT2; **(C)** STAT3; **(D)** STAT4; **(E)** STAT5A; **(F)** STAT5B; **(G)** STAT6. *p < 0.05, **p < 0.01, ***p < 0.001.

Scatterplot analysis revealed Spearman’s correlation coefficient between each STAT family gene and the tumor infiltration of each cell type analyzed in different cancer types (**Figures 9A-G**). STAT1 expression positively correlated with the infiltration of T regulatory cells (Tregs), T follicular helper cells (TFHs), CD8+ and activated memory CD4+ T cells, M1 macrophages, resting dendritic cells (DCs), and naive B cells in most tumors and negatively related to the infiltration of naive CD4+ T, plasma, resting NK, monocyte, resting and activated mast cells, M2 and M0 macrophages, activated DCs, and memory B cells in the majority of tumors (**Figure 9A**). Infiltration of resting memory CD4+ T cells and M1 macrophages positively associated with STAT2 expression; however, STAT2 negatively correlated with the infiltration of activated mast and memory B cells in most cancer types (**Figure 9B**). STAT3 expression positively correlated with the infiltration of resting memory CD4+ T and naive B cells in most tumors and negatively associated with the infiltration of TFHs, CD8+ T, activated NK, and memory B cells in different cancer types (**Figure 9C**). Infiltration of Tregs, TFHs, CD8+, resting and activated memory CD4+ T cells, M1 macrophages, resting DCs, and naive B cells positively correlated with STAT4 expression; however, STAT4 negatively correlated with the infiltration of naive CD4+ T, resting NK and resting mast cells, M0 macrophages, activated DCs, and memory B cells in most tumor types (**Figure 9D**). STAT5A expression positively correlated with the infiltration of Tregs, CD8+, resting and activated memory CD4+ T cells, M1 macrophages, resting DCs, and naive B cells in most tumors and negatively correlated with the infiltration of naive CD4+ T cells, M0 macrophages, activated DCs, and memory B cells in the majority of tumors (**Figure 9E**). The levels of STAT5B positively associated with the infiltration of resting memory CD4+ T, resting mast, and naive B cells in most tumors; however, STAT5B negatively correlated with the infiltration of Tregs, gammadelta T cells, TFHs, activated NK cells, and memory B cells in different cancer types (**Figure 9F**). STAT6 expression positively associated with the infiltration of resting memory CD4+ T, monocyte, and resting mast cells in most tumors and negatively related to the infiltration of gammadelta T cells, activated memory CD4+ T cells, and M0 macrophages in the majority of tumors (**Figure 9G**).

### TME analysis

**Figure 10:**
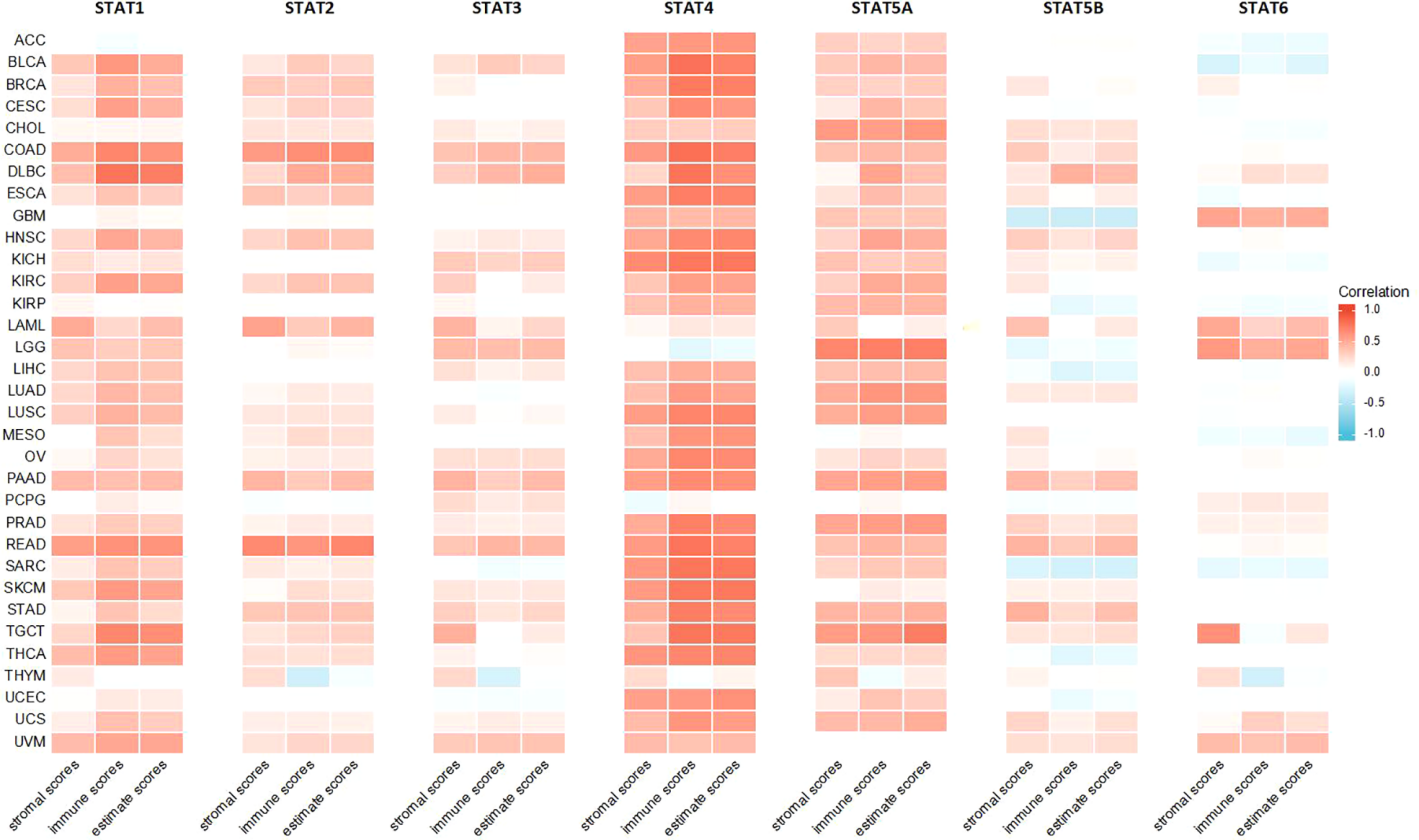
Association of the STAT family gene expression with stromal scores, immune scores, and estimate scores across 33 different cancer types.

Higher stromal or immune scores indicated that stromal or immune elements have infiltrated deeper into the TME. Among all STAT genes, but not STAT6, expression was positively related to stromal scores. Specifically, STAT1 was strongly correlated with READ, LAML, and COAD. STAT2 was strongly correlated with COAD, LAML, and READ. STAT3 was strongly correlated with TGCT, PAAD, and LAML. STAT4 was strongly correlated with ACC, BLCA, BRCA, COAD, ESCA, HNSC, KICH, LUSC, OV, PAAD, PRAD, READ, SARC, SKCM, THCA, and UCEC. STAT5A was strongly correlated with CHOL, LGG, LUAD, PAAD, PRAD, and TGCT. STAT5B was strongly correlated with PAAD, READ, and STAD, and STAT6 was strongly correlated with GBM and TGCT. The expressions of STAT1, STAT2, STAT3, STAT4, and STAT5A were mostly positively related to immune scores, and STAT5B and STAT6 expressions were partly positively related to immune scores. To be specific, STAT1 was closely related to BLCA, CESC, COAD, DLBC, HNSC, KIRC, READ, SKCM, TGCT, THCA, and UVM. STAT2 was closely related to COAD, DLBC, and READ. STAT3 was closely related to COAD, DLBC, and READ. STAT4 was closely related to ACC, BLCA, BRCA, CESC, COAD, DLBC, ESCA, HNSC, KICH, KIRC, LUAD, LUSC, MESO, OV, PAAD, PRAD, READ, SARC, SKCM, STAD, TGCT, THCA, UCEC, and UCS. STAT5A was closely related to CHOL, DLBC, HNSC, KIRC, LUAD, LUSC, PAAD, PRAD, and TGCT. STAT5B was positively correlated with DLBC but negatively related to GBM and SARC. All STAT genes except STAT6 exhibited a positive correlation with estimate scores. In particular, STAT1 was highly associated with BLCA, COAD, DLBC, KIRC, READ, SKCM, TGCT, THCA, and UVM. STAT2 was highly associated with COAD and READ. STAT3 was highly associated with COAD, DLBC, LGG, PAAD, and READ. STAT4 was highly associated with ACC, BLCA, BRCA, CESC, COAD, DLBC, ESCA, HNSC, KICH, KIRC, LUAD, LUSC, MESO, OV, PAAD, PRAD, READ, SARC, SKCM, STAD, TGCT, THCA, UCEC, and UCS. STAT5A was highly associated with CHOL, LUAD, LUSC, PAAD, PRAD, and TGCT. STAT5B was highly associated with READ and DLBC (**Table 1**; **Figure 10**).

**Table 1:** Association of the STAT family gene expression with stromal scores, immune scores and estimate scores across 33 different cancer types.

			ACC	BLCA	BRCA	CESC	CHOL	COAD	DLBC	ESCA	GBM	HNSC	KICH	KIRC	KIRP	LAML	LGG	LIHC	LUAD	LUSC	MESO	OV	PAAD	PCPG	PRAD	READ	SARC	SKCM	STAD	TGCT	THCA	THYM	UCEC	UCS	UVM
STAT1	stromal scores	r	0.01	0.35	0.20	0.23	0.09	0.50	0.40	0.21	0.00	0.26	0.23	0.30	0.10	0.51	0.38	0.26	0.26	0.31	0.00	0.09	0.41	-0.02	0.20	0.57	0.15	0.35	0.11	0.27	0.41	0.16	0.06	0.14	0.41
		P	9.03E-01	7.19E-13	1.08E-10	9.47E-05	6.16E-01	1.38E-19	6.45E-03	3.68E-03	9.79E-01	2.91E-09	6.94E-02	1.94E-12	9.89E-02	1.05E-15	3.29E-19	5.74E-07	2.35E-09	1.54E-12	9.69E-01	6.49E-02	1.82E-08	7.82E-01	4.80E-06	4.05E-09	1.75E-02	1.91E-14	2.52E-02	1.58E-03	1.59E-21	8.78E-02	3.90E-01	3.06E-01	1.60E-04
	immune scores	r	-0.11	0.61	0.47	0.56	0.09	0.70	0.79	0.37	0.11	0.53	0.16	0.56	0.04	0.26	0.32	0.38	0.44	0.47	0.37	0.28	0.39	0.15	0.33	0.64	0.38	0.60	0.37	0.69	0.59	-0.06	0.17	0.39	0.53
		P	3.41E-01	5.94E-43	1.90E-59	3.18E-25	5.86E-01	8.61E-43	7.56E-11	2.59E-07	1.80E-01	4.23E-39	2.01E-01	5.55E-45	5.37E-01	1.07E-04	7.42E-14	1.23E-13	6.18E-25	6.14E-28	4.64E-04	6.67E-09	8.77E-08	4.58E-02	2.53E-14	5.97E-12	2.63E-10	2.45E-46	7.01E-14	3.74E-20	8.69E-48	5.11E-01	2.76E-02	3.04E-03	4.43E-07
	estimate scores	r	-0.05	0.50	0.39	0.46	0.10	0.63	0.74	0.31	0.07	0.44	0.20	0.52	0.06	0.40	0.35	0.36	0.39	0.42	0.23	0.21	0.41	0.08	0.30	0.63	0.32	0.54	0.25	0.66	0.55	0.00	0.15	0.32	0.53
		P	6.56E-01	1.98E-27	8.90E-41	1.48E-16	5.69E-01	1.00E-32	4.01E-09	1.79E-05	3.88E-01	2.17E-26	1.08E-01	2.92E-38	3.26E-01	1.72E-09	4.19E-16	2.86E-12	1.83E-19	4.04E-22	3.05E-02	1.32E-05	9.46E-09	2.73E-01	1.57E-11	2.88E-11	1.25E-07	3.85E-36	5.30E-07	3.92E-18	1.46E-40	9.74E-01	4.28E-02	1.61E-02	6.30E-07
STAT2	stromal scores	r	0.00	0.18	0.33	0.19	0.20	0.59	0.26	0.37	0.04	0.27	0.03	0.26	0.03	0.55	0.04	0.03	0.10	0.18	0.14	0.13	0.45	-0.10	0.11	0.68	0.17	0.07	0.35	0.19	0.22	0.24	0.02	0.14	0.20
		P	9.91E-01	2.93E-04	1.21E-28	8.45E-04	2.47E-01	3.73E-28	8.29E-02	2.15E-07	6.66E-01	2.66E-10	8.10E-01	9.71E-10	6.46E-01	9.86E-19	4.15E-01	5.33E-01	1.73E-02	4.34E-05	1.86E-01	6.61E-03	2.52E-10	1.78E-01	1.68E-02	6.96E-14	5.02E-03	1.60E-01	7.66E-13	3.14E-02	3.04E-07	9.23E-03	7.37E-01	2.98E-01	7.72E-02
	immune scores	r	-0.01	0.33	0.31	0.30	0.19	0.66	0.51	0.31	0.07	0.39	0.01	0.37	-0.01	0.33	0.10	0.01	0.17	0.20	0.25	0.15	0.33	-0.06	0.16	0.62	0.13	0.24	0.37	0.26	0.21	-0.34	-0.04	0.14	0.28
		P	9.19E-01	9.88E-12	4.13E-25	2.00E-07	2.63E-01	2.10E-36	2.90E-04	1.64E-05	4.04E-01	4.28E-20	9.00E-01	7.45E-19	8.45E-01	8.11E-07	3.33E-02	7.72E-01	9.95E-05	8.72E-06	2.24E-02	2.09E-03	5.33E-06	3.87E-01	4.56E-04	4.28E-11	3.47E-02	1.65E-07	7.10E-14	2.48E-03	1.51E-06	1.96E-04	6.50E-01	2.94E-01	1.10E-02
	estimate scores	r	-0.01	0.27	0.36	0.29	0.19	0.66	0.49	0.37	0.06	0.37	0.03	0.37	0.00	0.45	0.07	0.03	0.15	0.20	0.22	0.16	0.42	-0.08	0.15	0.69	0.17	0.19	0.38	0.30	0.23	-0.12	0.00	0.13	0.27
		P	9.45E-01	4.75E-08	1.49E-34	5.52E-07	2.56E-01	1.75E-36	5.01E-04	3.06E-07	4.91E-01	2.07E-18	8.23E-01	1.66E-18	9.50E-01	3.73E-12	1.04E-01	6.17E-01	4.81E-04	7.12E-06	4.37E-02	9.48E-04	8.19E-09	3.02E-01	1.11E-03	3.85E-14	5.65E-03	4.06E-05	3.52E-15	4.67E-04	1.01E-07	1.77E-01	9.55E-01	3.22E-01	1.47E-02
STAT3	stromal scores	r	-0.01	0.21	0.13	-0.02	0.17	0.38	0.31	0.02	0	0.12	0.34	0.31	0.1	0.46	0.42	0.22	0.04	0.15	-0.01	0.22	0.46	0.25	0.16	0.36	-0.04	0.19	0.31	0.48	0.12	0.26	-0.1	0.11	0.33
		P	9.54E-01	1.24E-05	1.40E-05	7.83E-01	3.15E-01	4.03E-11	3.69E-02	8.03E-01	9.82E-01	5.32E-03	5.50E-03	5.40E-13	9.16E-02	9.16E-13	1.27E-22	2.15E-05	3.32E-01	6.15E-04	9.11E-01	3.62E-06	7.66E-11	8.29E-04	3.94E-04	3.82E-04	4.70E-01	3.80E-05	2.47E-10	4.59E-09	7.79E-03	3.93E-03	1.77E-01	4.36E-01	2.90E-03
	immune scores	r	-0.01	0.33	-0.05	0.02	0.09	0.44	0.44	0.05	-0.01	0.17	0.28	0.03	-0.06	0.11	0.4	0.12	-0.1	0.05	-0.06	0.22	0.31	0.16	0.13	0.45	-0.14	0.15	0.19	-0.06	0.04	-0.3	-0.12	0.15	0.37
		P	9.44E-01	8.87E-12	9.13E-02	7.56E-01	5.88E-01	4.19E-15	2.08E-03	5.16E-01	9.03E-01	9.00E-05	2.51E-02	5.34E-01	3.49E-01	1.14E-01	3.39E-21	2.56E-02	2.45E-02	2.38E-01	5.65E-01	5.40E-06	2.05E-05	2.86E-02	3.63E-03	8.30E-06	2.25E-02	1.34E-03	1.16E-04	4.64E-01	4.05E-01	8.24E-04	1.17E-01	2.73E-01	8.60E-04
	estimate scores	r	0	0.28	0.03	0.01	0.14	0.44	0.49	0.03	0.01	0.16	0.32	0.16	0.01	0.27	0.42	0.17	-0.04	0.1	-0.05	0.24	0.41	0.22	0.15	0.43	-0.11	0.18	0.27	0.16	0.07	-0.08	-0.11	0.13	0.38
		P	9.70E-01	5.81E-09	3.56E-01	8.83E-01	4.04E-01	1.41E-14	5.50E-04	6.41E-01	9.22E-01	1.93E-04	1.01E-02	1.89E-04	9.26E-01	7.25E-05	1.00E-22	9.88E-04	3.92E-01	2.37E-02	6.30E-01	5.26E-07	1.40E-08	3.81E-03	5.79E-04	2.22E-05	8.08E-02	1.22E-04	6.62E-08	6.69E-02	1.04E-01	3.80E-01	1.27E-01	3.35E-01	4.92E-04
STAT4	stromal scores	r	0.55	0.57	0.50	0.35	0.32	0.60	0.27	0.58	0.45	0.52	0.67	0.36	0.37	0.10	-0.03	0.39	0.40	0.58	0.39	0.51	0.57	-0.16	0.50	0.59	0.61	0.59	0.49	0.37	0.62	0.25	0.56	0.42	0.41
		P	1.66E-07	1.12E-35	3.05E-70	7.93E-10	6.13E-02	1.46E-28	6.49E-02	1.65E-17	8.21E-09	3.48E-37	9.53E-10	4.79E-18	1.71E-10	1.27E-01	4.61E-01	5.50E-15	1.06E-20	2.49E-46	2.02E-04	2.26E-29	2.40E-16	2.87E-02	2.22E-33	7.41E-10	3.49E-27	1.13E-43	4.97E-25	1.13E-05	9.77E-55	7.26E-03	7.39E-16	1.44E-03	2.10E-04
	immune scores	r	0.61	0.80	0.76	0.67	0.31	0.81	0.79	0.74	0.42	0.68	0.77	0.57	0.47	0.18	-0.23	0.49	0.60	0.71	0.63	0.69	0.67	0.10	0.73	0.79	0.78	0.77	0.74	0.78	0.69	-0.08	0.60	0.62	0.38
		P	3.98E-09	5.93E-92	1.60E-201	1.92E-39	6.97E-02	1.58E-66	5.53E-11	7.95E-33	7.08E-08	1.74E-70	1.08E-13	4.90E-47	3.69E-17	7.31E-03	2.14E-07	2.16E-23	1.79E-49	1.83E-76	1.24E-10	3.09E-61	1.38E-24	2.01E-01	9.45E-84	9.29E-21	2.24E-53	3.66E-91	1.01E-69	4.52E-28	2.38E-71	3.98E-01	5.37E-19	3.66E-07	5.98E-04
	estimate scores	r	0.62	0.72	0.73	0.60	0.32	0.74	0.65	0.71	0.44	0.69	0.77	0.55	0.45	0.17	-0.16	0.48	0.54	0.69	0.63	0.67	0.65	-0.03	0.68	0.72	0.77	0.76	0.67	0.76	0.70	0.11	0.64	0.58	0.41
		P	1.48E-09	6.02E-66	8.24E-177	1.17E-29	5.80E-02	6.71E-50	9.42E-07	1.43E-29	1.51E-08	6.98E-73	1.08E-13	2.62E-43	6.30E-16	1.45E-02	2.29E-04	2.81E-22	1.27E-39	2.04E-70	1.50E-10	6.77E-55	8.33E-23	6.47E-01	3.49E-67	1.43E-15	1.93E-51	1.04E-84	1.26E-52	1.78E-26	7.31E-75	2.26E-01	3.82E-22	2.52E-06	1.88E-04
STAT5A	stromal scores	r	0.31	0.33	0.30	0.16	0.59	0.38	0.09	0.20	0.36	0.28	0.38	0.30	0.41	0.34	0.69	0.37	0.50	0.49	-0.07	0.19	0.53	-0.05	0.53	0.38	0.27	0.03	0.45	0.58	0.25	0.36	0.15	0.41	0.02
		P	5.33E-03	1.41E-11	1.16E-23	7.68E-03	1.44E-04	5.18E-11	5.60E-01	7.45E-03	5.42E-06	6.34E-11	2.03E-03	2.85E-12	8.42E-13	4.38E-07	1.33E-71	4.33E-13	1.93E-32	2.90E-31	5.08E-01	1.38E-04	3.14E-14	4.80E-01	2.23E-37	2.46E-04	8.18E-06	4.68E-01	2.82E-20	1.88E-13	1.12E-08	6.09E-05	3.92E-02	1.83E-03	8.30E-01
	immune scores	r	0.28	0.44	0.29	0.44	0.57	0.43	0.54	0.41	0.35	0.54	0.32	0.51	0.46	-0.01	0.73	0.40	0.62	0.58	0.10	0.29	0.57	0.10	0.58	0.45	0.35	0.16	0.44	0.62	0.26	-0.14	0.37	0.43	-0.03
		P	1.37E-02	2.76E-20	3.31E-22	2.55E-15	2.62E-04	7.38E-14	1.25E-04	1.29E-08	1.01E-05	1.06E-39	8.30E-03	1.48E-36	3.96E-16	8.36E-01	1.27E-84	1.20E-15	4.26E-54	1.60E-45	3.84E-01	9.14E-10	8.85E-17	2.04E-01	1.33E-45	7.14E-06	5.09E-09	6.01E-04	9.18E-20	2.66E-15	5.04E-09	1.27E-01	4.62E-07	8.89E-04	7.69E-01
	estimate scores	r	0.31	0.41	0.34	0.35	0.61	0.42	0.39	0.33	0.36	0.48	0.36	0.49	0.45	0.14	0.73	0.42	0.61	0.57	0.01	0.27	0.58	0.04	0.60	0.42	0.36	0.12	0.48	0.74	0.26	0.15	0.31	0.49	0.00
		P	6.90E-03	1.59E-17	5.04E-30	5.00E-10	6.83E-05	1.70E-13	7.84E-03	4.89E-06	4.81E-06	5.31E-31	3.46E-03	4.26E-33	6.21E-16	3.57E-02	1.25E-85	2.67E-17	4.18E-52	1.46E-43	9.53E-01	3.29E-08	1.52E-17	6.01E-01	2.84E-50	4.23E-05	2.44E-09	8.00E-03	6.87E-24	2.01E-24	1.73E-09	1.07E-01	2.14E-05	1.16E-04	9.89E-01
STAT5B	stromal scores	r	0.04	0.03	0.18	0.03	0.24	0.33	0.18	0.20	-0.36	0.32	0.16	0.18	-0.06	0.39	-0.21	-0.12	0.17	0.02	0.20	0.14	0.43	-0.11	0.30	0.46	-0.28	0.13	0.47	0.19	-0.09	0.11	-0.03	0.28	0.23
		P	7.03E-01	5.36E-01	1.38E-09	5.99E-01	1.58E-01	1.68E-08	2.44E-01	5.64E-03	6.75E-06	3.94E-14	2.11E-01	2.59E-05	3.01E-01	5.03E-09	2.88E-06	2.74E-02	1.33E-04	6.04E-01	6.51E-02	5.61E-03	3.71E-09	1.31E-01	8.20E-12	4.76E-06	4.39E-06	7.25E-03	5.35E-23	2.60E-02	3.37E-02	2.35E-01	7.02E-01	3.61E-02	4.51E-02
	immune scores	r	0.06	-0.03	-0.05	-0.08	0.19	0.16	0.47	0.06	-0.37	0.20	0.09	-0.09	-0.21	0.01	-0.11	-0.26	0.17	-0.03	-0.07	0.02	0.30	-0.10	0.18	0.34	-0.32	0.13	0.25	0.19	-0.24	-0.05	-0.18	0.13	0.20
		P	5.85E-01	5.88E-01	1.20E-01	1.49E-01	2.74E-01	7.68E-03	1.03E-03	4.10E-01	1.95E-06	5.10E-06	4.55E-01	2.96E-02	2.81E-04	8.78E-01	1.13E-02	3.77E-07	2.05E-04	4.62E-01	5.08E-01	7.36E-01	3.70E-05	1.95E-01	4.84E-05	9.39E-04	2.06E-07	5.44E-03	5.20E-07	3.34E-02	8.11E-08	5.60E-01	1.94E-02	3.51E-01	7.31E-02
	estimate scores	r	0.06	0.00	0.07	-0.03	0.21	0.26	0.41	0.15	-0.37	0.29	0.13	0.02	-0.16	0.17	-0.15	-0.22	0.19	-0.01	0.04	0.08	0.39	-0.10	0.25	0.43	-0.32	0.14	0.39	0.24	-0.19	0.05	-0.12	0.22	0.24
		P	5.76E-01	9.77E-01	2.44E-02	6.02E-01	2.24E-01	6.58E-06	4.93E-03	4.73E-02	3.47E-06	1.10E-11	3.13E-01	6.86E-01	5.40E-03	1.21E-02	6.90E-04	3.45E-05	2.76E-05	8.14E-01	7.24E-01	8.40E-02	6.70E-08	1.74E-01	1.05E-08	1.65E-05	1.04E-07	2.66E-03	1.04E-15	5.53E-03	1.51E-05	6.24E-01	1.13E-01	9.70E-02	3.28E-02
STAT6	stromal scores	r	-0.12	-0.30	0.13	-0.10	-0.06	0.00	0.08	-0.13	0.54	0.01	-0.17	0.03	-0.09	0.52	0.60	0.04	-0.08	-0.08	-0.15	0.00	-0.06	0.14	0.13	0.05	-0.21	-0.05	-0.03	0.64	0.01	0.24	-0.07	0.08	0.42
		P	2.95E-01	5.36E-10	2.43E-05	1.03E-01	7.37E-01	9.49E-01	6.14E-01	7.26E-02	1.09E-12	8.42E-01	1.84E-01	4.39E-01	1.34E-01	5.45E-16	9.59E-51	4.50E-01	7.25E-02	8.06E-02	1.61E-01	9.51E-01	4.17E-01	6.24E-02	3.15E-03	6.58E-01	8.91E-04	3.37E-01	5.26E-01	1.28E-16	8.97E-01	9.26E-03	3.88E-01	5.55E-01	1.39E-04
	immune scores	r	-0.19	-0.18	-0.03	-0.02	-0.11	0.07	0.23	0.01	0.47	0.07	-0.10	-0.02	-0.13	0.29	0.48	-0.09	0.06	-0.02	-0.14	0.08	0.03	0.18	0.13	0.10	-0.18	-0.07	-0.03	-0.11	-0.04	-0.33	-0.05	0.31	0.37
		P	9.85E-02	3.88E-04	3.20E-01	7.12E-01	5.12E-01	2.44E-01	1.26E-01	8.42E-01	1.01E-09	9.41E-02	4.16E-01	6.42E-01	2.33E-02	1.20E-05	8.87E-30	9.91E-02	1.96E-01	7.25E-01	1.95E-01	1.05E-01	6.71E-01	1.45E-02	5.35E-03	3.52E-01	2.95E-03	1.21E-01	5.56E-01	1.90E-01	3.45E-01	2.30E-04	4.72E-01	2.08E-02	6.96E-04
	estimate scores	r	-0.17	-0.26	0.05	-0.05	-0.11	0.03	0.21	-0.07	0.50	0.04	-0.13	-0.01	-0.12	0.41	0.53	-0.03	-0.01	-0.05	-0.20	0.05	-0.01	0.17	0.13	0.07	-0.20	-0.07	-0.04	0.18	-0.03	-0.09	-0.06	0.22	0.41
		P	1.42E-01	1.48E-07	1.20E-01	4.19E-01	5.38E-01	6.65E-01	1.61E-01	3.58E-01	3.30E-11	3.47E-01	2.85E-01	8.74E-01	3.55E-02	6.90E-10	3.22E-38	5.07E-01	7.92E-01	2.92E-01	7.05E-02	3.24E-01	8.67E-01	2.46E-02	3.41E-03	5.30E-01	1.35E-03	1.66E-01	4.54E-01	3.40E-02	5.39E-01	3.42E-01	4.39E-01	1.01E-01	1.80E-04

STAT, signal transducer and activator of transcription; ACC, adrenocortical carcinoma; BLCA, bladder urothelial carcinoma; BRCA, breast invasive carcinoma; CESC, cervical squamous cell carcinoma and endocervical adenocarcinoma; CHOL, cholangiocarcinoma; COAD, colon adenocarcinoma; DLBC, lymphoid neoplasm diffuse large B-cell lymphoma; ESCA, esophageal carcinoma; GBM, glioblastoma multiforme; HNSC, head and neck squamous cell carcinoma; KICH, kidney chromophobe; KIRC, kidney renal clear cell carcinoma; KIRP, kidney renal papillary cell carcinoma; LAML, acute myeloid leukemia; LGG, brain lower grade glioma; LIHC, liver hepatocellular carcinoma; LUAD, lung adenocarcinoma; LUSC, lung squamous cell carcinoma; MESO, mesothelioma; OV, ovarian serous cystadenocarcinoma; PAAD, pancreatic adenocarcinoma; PCPG, pheochromocytoma and paraganglioma; PRAD, prostate adenocarcinoma; READ, rectum adenocarcinoma; SARC, sarcoma; SKCM, skin cutaneous melanoma; STAD, stomach adenocarcinoma; TGCT, testicular germ cell tumors; THCA, thyroid carcinoma; THYM, thymoma; UCEC, uterine corpus endometrial carcinoma; UCS, uterine carcinosarcoma; UVM, uveal melanoma. The red values mean that the differences are statistically significant.

**Figure 11:**
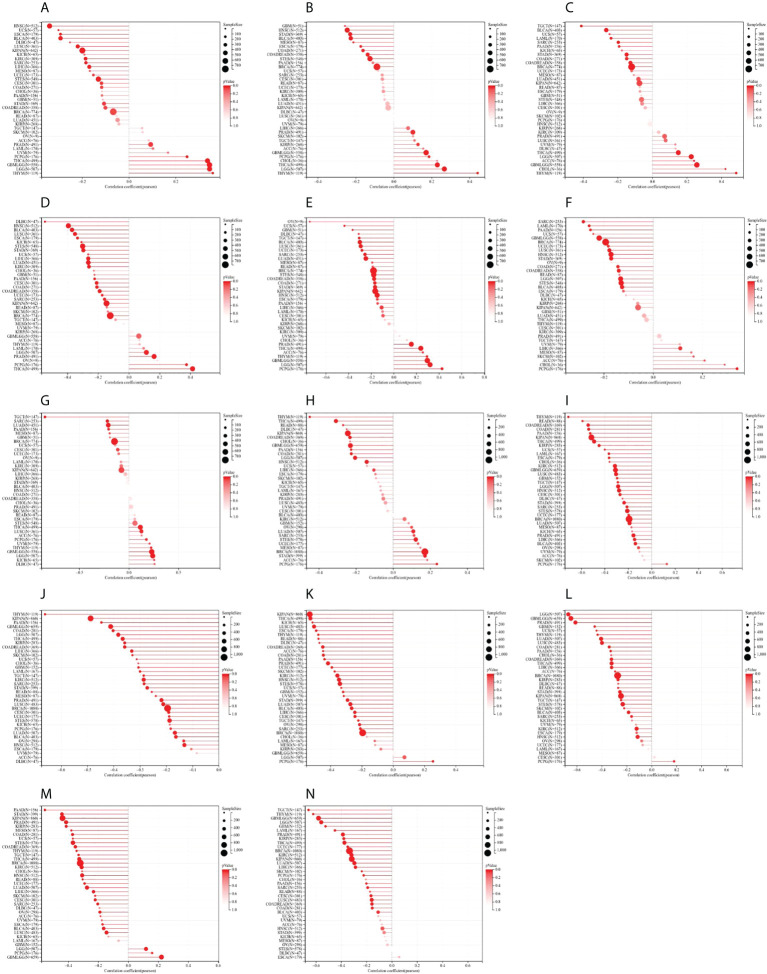
Correlation between signal transducer and activator of transcription (STAT) gene expression and cancer stemness scores based on Spearman’s correlation tests. **(A–G)** DNAss. **(H–N)** RNAss.

In DNAss, the expression of STAT1 was positively related to GBMLGG, LGG, PRAD, THYM, THCA, and PCPG, while STAT1 expression was negatively related to CESC, ESCA, Stomach and Esophageal carcinoma (STES), SARC, KIPAN, STAD, UCEC, HNSC, KIRC, LUSC, LIHC, UCS, and BLCA (**Figure 11A**). The expression of STAT2 was positively related to GBMLGG, LGG, KIRP, PRAD, THYM, THCA, and PCPG, while STAT2 expression was negatively related to COAD, COADREAD, BRCA, ESCA, STES, STAD, HNSC, MESO, and BLCA (**Figure 11B**). The expression of STAT3 was positively related to GBMLGG, LGG, THYM, THCA, ACC, and CHOL, while STAT3 expression was negatively related to COAD, COADREAD, LAML, BRCA, SARC, STAD, PAAD, TGCT, and BLCA (**Figure 11C**). The expression of STAT4 was positively related to LGG, PARD, THCA, and PCPG, while STAT4 expression was negatively related to CESC, LUAD, COAD, ESCA, COADREAD, BRCA, STES, SARC, HNSC, KIPAN, STAD, UCEC, KIRC, LUSC, LIHC, PAAD, UCS, BLCA, KICH, and DLBC (**Figure 11D**). The expression of STAT5A was positively related to GBMLGG, LGG, PARD, THYM, THCA, PCPG, and ACC, while STAT5A expression was negatively related to GBM, LUAD, COAD, UCEC, COADREAD, BRCA, STES, SARC, KIPAN, STAD, HNSC, LUSC, LIHC, OV, TGCT, UCS, BLCA, and DLBC (**Figure 11E**). The expression of STAT5B was positively related to LIHC and PCPG, while STAT5B expression was negatively related to GBMLGG, LGG, COAD, LAML, COADREAD, BRCA, STES, SARC, STAD, UCEC, HNSC, LUSC, PAAD, and BLCA (**Figure 11F**). The expression of STAT6 was positively related to GBMLGG, LGG, LUSC, THYM, THCA, PCPG, and KICH, while STAT6 expression was negatively correlated with LUAD, BRCA, SARC, PAAD, and TGCT (**Figure 11G**).

In RNAss, the expression of STAT1 was positively associated with LUAD, BRCA, STES, STAD, and PCPG, while STAT1 expression was negatively associated with GBMLGG, LGG, COAD, COADREAD, KIPAN, HNSC, THYM, LIHC, THCA, READ, and PAAD (**Figure 11H**). The expression of STAT2 was almost negatively associated with all tumor types except ACC, SKCM, and PCPG (**Figure 11I**). The expression of STAT3 was negatively associated with all tumor types (**Figure 11J**). The expression of STAT4 was positively associated with PCPG, while STAT4 expression was negatively related to all other tumor types (**Figure 11K**). The expression of STAT5A was positively associated with PCPG, while STAT5A expression was negatively related to all other tumor types (**Figure 11L**). The expression of STAT5B was positively associated with GBMLGG, LGG, and PCPG, while STAT5B expression was negatively related to all other tumor types (**Figure 11M**). The expression of STAT6 was negatively associated with GBM, GBMLGG, LGG, CESC, LUAD, COAD, COADREAD, LAML, BRCA, SARC, KIRP, KIPAN, PRAD, UCEC, KIRC, LUSC, THYM, LIHC, THCA, PAAD, TGCT, PCPG, SKCM, and BLCA (**Figure 11N**).

### PPI network construction

**Figure 12:**
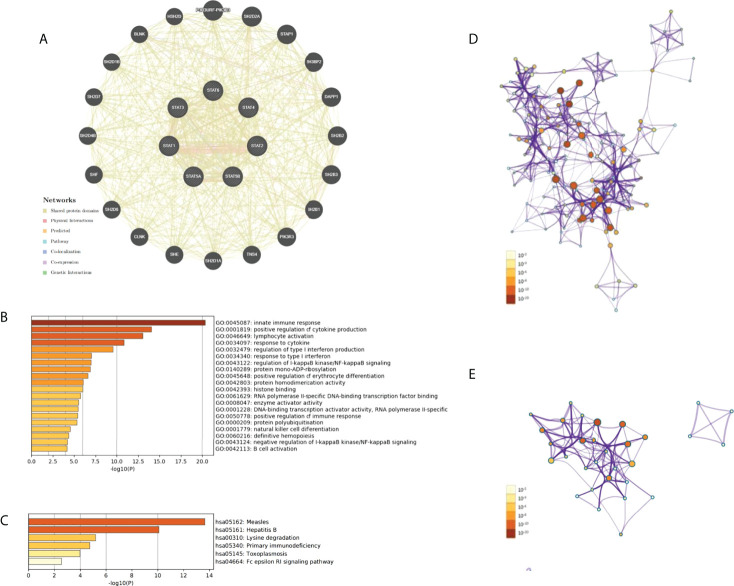
PPI network, GO and KEGG enrichment analysis in pan-cancer. **(A)** The PPI network for STATs and the 20 most frequently altered neighbor genes. **(B)** The network of enriched GO terms. **(C)** The network of enriched KEGG terms. **(D)** The network of GO pathways colored by p-value. **(E)** The network of KEGG pathways colored by p-value.

We constructed a PPI network for each of the STAT genes using the GeneMANIA database. A hub node representing each member of the STAT family was surrounded by 20 nodes corresponding to genes that were significantly correlated with their members (**Figure 12A**).

### GO and KEGG enrichment analysis

GEPIA was used to identify the top 20 genes that are similar to those of each STAT member in order to explore possible mechanisms through which STATs can contribute to pan-cancer pathology (**Table 2**). After removing duplicates, there were 135 genes, including seven STAT family factors and 128 similar genes. To catch the relationship between terms, the Metascape database was used in GO and KEGG enrichment analyses of STATs and their relative genes. Three aspects, including MF, CC, and BP, were considered in GO enrichment analysis for predicting target host gene functions. As displayed in **Figure 12B**, we found that GO:0045087 (innate immune response), GO:0001819 (positive regulation of cytokine production), GO:0046649 (lymphocyte activation), GO:0034097 (response to cytokine), GO:0032479 (regulation of type I interferon production), GO:0034340 (response to type I interferon), GO:0043122 (regulation of I-kappaB kinase/NF-kappaB signaling), GO:0140289 (protein mono-ADP-ribosylation), GO:0045648 (positive regulation of erythrocyte differentiation), GO:0042803 (protein homodimerization activity), GO:0042393 histone binding, GO:0061629 (RNA polymerase II-specific DNA-binding transcription factor binding), GO:0008047 (enzyme activator activity), GO:0001228 (DNA-binding transcription activator activity, RNA polymerase II-specific), GO:0050778 (positive regulation of immune response), GO:0000209 (protein polyubiquitination), GO:0001779 (NK cell differentiation), GO:0060216 (definitive hemopoiesis), GO:0043124 (negative regulation of I-kappaB kinase/NF-kappaB signaling), and GO:0042113 (B cell activation) were prominently related to STAT factors and their similar genes. Furthermore, six pathways related to the functions of the STAT family were found through KEGG analysis; pathways such as hsa05162: measles, hsa05161: hepatitis B, hsa00310: lysine degradation, hsa05340: primary immunodeficiency, hsa05145: toxoplasmosis, and hsa04664: Fc epsilon RI signaling pathway were involved in the tumorigenesis and pathogenesis of pan-cancer (**Figure 12C**). Additionally, the networks of enriched GO terms and KEGG pathways were displayed in **Figures 12D, E**.

**Table 2:** The top 20 similar genes of each STAT family gene.

STAT1	STAT2	STAT3	STAT4	STAT5A	STAT5B	STAT6
TAP1	XAF1	TRIP12	AC067945.4	NRROS	EZH1	TRIM38
UBE2L6	TRIM22	GOSR1	SLFN12L	FAM78A	LRRC37A17P	SP1
IFIH1	PARP14	SPTY2D1	RP11-1094M14.5	STK10	KMT2A	CASP8
LAP3	SP110	MAP3K2	RP11-686D22.10	NCKAP1L	CARF	NUMB
PARP14	STAT1	KPNA6	RP11-1094M14.8	DOCK2	PIKFYVE	BAZ2A
APOL6	SAMD9L	MFAP3	LINC00861	RIN3	SENP7	IRAK4
GBP1	SP100	FBXW2	NLRC3	TMEM106A	ZNRD1-AS1	PLEKHM1P
PARP9	OAS2	SEC24B	SLA2	SPN	NKTR	RREB1
OAS2	ZFYVE26	EFCAB14	TOMM20P2	TMC8	TSSK4	ARHGAP27
DTX3L	BTN3A1	ACBD3	SAMD3	INPP5D	CTD-2647L4.4	ALPK1
OAS3	RNF213	STX17	AKNA	GIT2	CREBRF	NONOP2
IRF1	ADAR	STAM2	CD40LG	PIK3CD	ZZEF1	KMT2D
TAP2	OAS3	SUSD6	LY9	CARD8	NR2C2	GMIP
SAMD9L	DTX3L	ASXL2	RASAL3	LYL1	KANSL1	ELF1
GBP1P1	BAZ2A	SLAIN2	PARP15	SETDB2	GIT2	TRIM56
EPSTI1	PARP12	THRAP3	E2F3P1	FMNL1	FAM13B	RP11-295D4.3
DDX60	FKBP15	ADAR	GVINP1	GAB3	ZNF445	PHYKPL
TRIM21	MX2	SP3	CD6	RRN3P3	TTBK2	GIT2
IFIT3	PARP9	RBM12	SLAMF6	BTK	PARGP1	RP11-274B21.2
GBP5	IFIH1	SEC23IP	CLEC2D	RP4-682C21.2	PAPD4	ITSN2

## Discussion

In many malignancies, a dysregulation of STAT family genes has been reported, but the value of STATs in the tumorigenesis and prognosis of a number of cancers has been partly established. In the present study, the gene expression and survival data, gene variation, methylation status, pathway activity, and drug sensitivity, as well as analyses of immune cell infiltration, identified STAT members as potential biomarkers, with great significance for pan-cancer research.

TCGA pan-cancer data showed that STAT family genes were extensively changed in BLCA, BRCA, CHOL, COAD, ESCA, HNSC, KICH, KIRC, KIRP, LIHC, LUAD, LUSC, STAD, THCA, and UCEC compared to the matched adjacent normal tissues. The survival plots revealed that high expressions of STAT1, STAT2, STAT3, STAT5A, and STAT6 were risk factors in GBMLGG and LGG; the higher expression of STAT1, STAT2, STAT3, STAT4, STAT5B, and STAT6 correlated with the lower OS rates in LAML. In KIPAN, upregulated STAT1, STAT2, STAT3, and STAT4 were linked to the shortened OS. High expression of STAT1, STAT3, and STAT6 correlated with better OS in UVM. However, the expression of STAT1, STAT3, STAT4, STAT5A, and STAT5B was a protective factor in SKCM. STAT1 and STAT4 were correlated with a high clinical prognosis. We also found that lower STAT5B expression was significantly correlated with poor prognosis in GBMLGG, which was different from other STATs. Thus, the STAT family was a prognostic biomarker in many cancers, with special emphasis on GBMLGG, LGG, LAML, KIPAN, UVM, and SKCM, which has not been previously reported.

A high frequency of CNV was found for STAT family genes. Expression analysis for STAT regulators showed a positive correlation between CNV and expression, particularly for STAT5B, STAT6, and STAT3. In addition, CNVs could affect STAT gene expression, a process that can cause tumorigenesis. In one example, the *STAT2* gene was often amplified in KICH, KIRC, and KIRP (all heterozygous amplification >25%, p < 0.05) and was related to a lower patient survival in *KIPAN*.

It was found that STAT methylation varied significantly among PRAD, LUSC, BRCA, UCEC, COAD, THCA, KIRC, and LIHC. According to a survival analysis of various types of tumors, hypermethylated STAT5A and STAT5B were mainly associated with poorer survival while hypomethylated STAT1, STAT2, and STAT4 were mainly associated with shorter survival. It is the first time that a change in STAT methylation status has been expected to predict survival outcomes.

An interaction network of STATs was linked to activation of cell apoptosis, EMT, hormone ER, and RAS/MAPK pathway and inhibition of cell cycle, DNA damage response, and hormone AR. Thus, STAT factors promoted tumorigenesis *via* a variety of mechanisms. Chemotherapy and target therapy clinical results were affected by molecular abnormalities. According to the sensitivity analysis, STAT5A and STAT5B at low levels showed high resistance to several drugs, indicating that they might serve as biomarkers for screening drugs. To take a few examples, I-BET762 has been used in PAAD and COADREAD (38, 39); navitoclax has been enrolled in phase II clinical trials (40, 41). Hence, these drugs are susceptible to anticancer effects *via* STAT regulation.

Functional enrichment analyses showed that STAT genes were functionally enriched in lymphocyte activation and immune response. In this study, STAT family members mainly influenced the infiltration of Tregs, CD8+, resting memory CD4+ T cells, M1 macrophages, and naive B cells and positively related to the infiltration of naive CD4+ T and NK cells, M0 macrophages, activated DCs, and memory B cells in most STAT family genes. In tumor immunity, STATs also played an important role. The relationship between immunity infiltration and expression of STAT family genes was also discussed. It was not unexpected that STATs were significantly influenced by the abundance of B, CD8+ and CD4+ T cells, macrophages, and DCs in the infiltration. Many published articles have shown that pSTAT3 acted to negatively affect T cells and DCs while positively regulating Tregs (42–45). STAT4 specifically mediated IL-12 signaling, affecting a wide range of immune cells. The biologic effects of IL-12 included induction of interferon expression in NK and activated T cells, increase in cytotoxic responses in both T and NK cells, and induction of proliferation of activated T cells (46, 47). By signaling with IL-4 and IL-13, STAT6 triggered an immune cell response, causing B- and T-cell proliferation and differentiation of macrophages (48, 49). In addition, the transcription factor STAT6 was the key to Th2 cell development as it decreased the production of IL-10 and increased IL-12 in DCs (50, 51). The study showed that STATs played a major role in tumor immune escape, as shown by the close correlation between STATs and immunocellular infiltration.

Based on the present study, STAT gene expression was positively affected by immune, stromal, and estimate scores in 33 tumors. According to the findings, the greater the number of immune and stromal cells, the greater the number of tumor cells. It was demonstrated that stromal and immune cells were involved in cancer growth, metastasis, and drug resistance, suggesting that STATs can regulate tumor behavior by interacting with the TME (52). In addition, most STAT gene expressions correlated positively with DNAss and RNAss in 33 tumors from TCGA. Increased expression of STATs, improved tumor stemness scores, stronger tumor stem cell activity, and decreased tumor differentiation were observed.

Despite these relevant strengths, it is nevertheless important to acknowledge the limitation of our study. Preclinical studies are expected to determine the influence of these immune-specific and tumor-specific STAT family genes on driving tumor infiltration and survival differences. Further biological experiments are needed to verify some important results in this study. We have collected tissue specimens of breast and cervical cancer, and in the future, validated experiments will be conducted to examine these findings.

In conclusion, considering these genomic alterations and clinical features of STAT family members across cancer types, it will be possible to change the relationship between STATs and tumorigenesis. It was beneficial to treat cancer by targeting these STAT regulators.

## Data availability statement

The datasets presented in this study can be found in online repositories. The names of the repository/repositories and accession number(s) can be found in the article/supplementary material.

## Author contributions

DC, YC, GS and MZ contributed to the conception and design of the study. MZ, PZ, MD drafted the manuscript. RY, YM, JZ, JX and TM collected and analyzed the data. All authors contributed to the article and approved the submitted version.

## Funding

This work was supported by the Natural Science Foundation of Qinghai Province (No. 2022-ZJ-912).

## Conflict of interest

The authors declare that the research was conducted in the absence of any commercial or financial relationships that could be construed as a potential conflict of interest.

The handling editor BH and reviewer MX declared a shared parent affiliation with the author(s) MZ, PZ, RY, TM, JX, YC, DC at the time of review.

## Publisher’s note

All claims expressed in this article are solely those of the authors and do not necessarily represent those of their affiliated organizations, or those of the publisher, the editors and the reviewers. Any product that may be evaluated in this article, or claim that may be made by its manufacturer, is not guaranteed or endorsed by the publisher.

## References

[1] Miklossy G Hilliard TS Turkson J. Therapeutic modulators of STAT signalling for human diseases. Nat Rev Drug Discovery (2013) 12(8):611–29. doi: 10.1038/nrd4088 PMC4038293 23903221

[2] Li C Cao Y Zhang L Li J Wu H Ling F. LncRNA IGFBP4-1 promotes tumor development by activating janus kinase-signal transducer and activator of transcription pathway in bladder urothelial carcinoma. Int J Biol Sci (2020) 16(13):2271–82. doi: 10.7150/ijbs.46986 PMC7378649 32760196

[3] Attarha S Reithmeier A Busker S Desroses M Page BDG. Validating signal transducer and activator of transcription (STAT) protein-inhibitor interactions using biochemical and cellular thermal shift assays. ACS Chem Biol (2020) 15(7):1842–51. doi: 10.1021/acschembio.0c00046 32412740

[4] Shahmarvand N Nagy A Shahryari J Ohgami RS. Mutations in the signal transducer and activator of transcription family of genes in cancer. Cancer Sci (2018) 109(4):926–33. doi: 10.1111/cas.13525 PMC5891179 29417693

[5] Benamu E. Infectious risks associated with biologics targeting janus kinase-signal transducer and activator of transcription signaling and complement pathway for inflammatory diseases. Infect Dis Clin North Am (2020) 34(2):271–310. doi: 10.1016/j.idc.2020.02.014 32444011

[6] Pruitt HC Metge BJ Weeks SE Chen D Wei S Kesterson RA. Conditional knockout of n-myc and STAT interactor disrupts normal mammary development and enhances metastatic ability of mammary tumors. Oncogene (2018) 37(12):1610–23. doi: 10.1038/s41388-017-0037-7 PMC5921859 29326438

[7] Jesser EA Brady NJ Huggins DN Witschen PM O'Connor CH Schwertfeger KL. STAT5 is activated in macrophages by breast cancer cell-derived factors and regulates macrophage function in the tumor microenvironment. Breast Cancer Res (2021) 23(1):104. doi: 10.1186/s13058-021-01481-0 34743736 PMC8573892

[8] Chan SR Vermi W Luo J Lucini L Rickert C Fowler AM. STAT1-deficient mice spontaneously develop estrogen receptor α-positive luminal mammary carcinomas. Breast Cancer Res (2012) 14(1):R16. doi: 10.1186/bcr3100 22264274 PMC3496133

[9] Zhang Y Zhang Y Yun H Lai R Su M. Correlation of STAT1 with apoptosis and cell-cycle markers in esophageal squamous cell carcinoma. PLoS One (2014) 9(12):e113928. doi: 10.1371/journal.pone.0113928 25438156 PMC4250046

[10] You W Tang Q Zhang C Wu J Gu C Wu Z. IL-26 promotes the proliferation and survival of human gastric cancer cells by regulating the balance of STAT1 and STAT3 activation. PLoS One (2013) 8(5):e63588. doi: 10.1371/journal.pone.0063588 23704922 PMC3660585

[11] Hosui A Klover P Tatsumi T Uemura A Nagano H Doki Y. Suppression of signal transducers and activators of transcription 1 in hepatocellular carcinoma is associated with tumor progression. Int J Cancer (2012) 131(12):2774–84. doi: 10.1002/ijc.27580 PMC3541944 22488367

[12] Widschwendter A Tonko-Geymayer S Welte T Daxenbichler G Marth C Doppler. Prognostic significance of signal transducer and activator of transcription 1 activation in breast cancer. Clin Cancer Res (2002) 8(10):3065–74. 12374673

[13] Khodarev N Ahmad R Rajabi H Pitroda S Kufe T McClary C. Cooperativity of the MUC1 oncoprotein and STAT1 pathway in poor prognosis human breast cancer. Oncogene (2010) 29(6):920–9. doi: 10.1038/onc.2009.391 PMC2820589 19915608

[14] Zimmerman MA Rahman NT Yang D Lahat G Lazar AJ Pollock RE. Unphosphorylated STAT1 promotes sarcoma development through repressing expression of fas and bad and conferring apoptotic resistance. Cancer Res (2012) 72(18):4724–32. doi: 10.1158/0008-5472.CAN-12-1347 PMC3564959 22805310

[15] Roeser JC Leach SD McAllister F. Emerging strategies for cancer immunoprevention. Oncogene (2015) 34(50):6029–39. doi: 10.1038/onc.2015.98 PMC11073473 26364615

[16] Yu H Pardoll D Jove R. STATs in cancer inflammation and immunity: A leading role for STAT3. Nat Rev Cancer (2009) 9(11):798–809. doi: 10.1038/nrc2734 19851315 PMC4856025

[17] Li CY Chen CY An JH Hutzen B Chan C Hsieh FC. Normal basal epithelial cells stimulate the migration and invasion of prostate cancer cell RM-1 by TGF-β1/STAT3 axis *in vitro* . Cancer Manag Res (2021) 13:3685–97. doi: 10.2147/CMAR.S303122 PMC8114913 33994809

[18] Liao PA Chu PY Tan ZL Axelrod JH Daum H Rottenberg Y. STAT3 inactivation and induction of apoptosis associate with fluoxetine-inhibited epithelial-mesenchymal transition and growth of triple-negative breast cancer *in vivo* . Anticancer Res (2022) 42(8):3807–14. doi: 10.21873/anticanres.15871 35896246

[19] Mo XT Leung TH Tang HW Yamaguchi T Terada A Fujiyoshi N. CD109 mediates tumorigenicity and cancer aggressiveness *via* regulation of EGFR and STAT3 signalling in cervical squamous cell carcinoma. Br J Cancer (2020) 123(5):833–43. doi: 10.1038/s41416-020-0922-7 PMC7463003 32507856

[20] Geiger JL Grandis JR Bauman JE. The STAT3 pathway as a therapeutic target in head and neck cancer: Barriers and innovations. Oral Oncol (2016) 56:84–92. doi: 10.1016/j.oraloncology.2015.11.022 26733183 PMC5590227

[21] Li S Priceman SJ Xin H Zhang W Deng J Liu Y. Icaritin inhibits JAK/STAT3 signaling and growth of renal cell carcinoma. PLoS One (2013) 8(12):e81657. doi: 10.1371/journal.pone.0081657 24324713 PMC3855768

[22] Wang Z Si X Xu A Meng X Gao S Qi Y. Activation of STAT3 in human gastric cancer cells *via* interleukin (IL)-6-type cytokine signaling correlates with clinical implications. PLoS One (2013) 8(10):e75788. doi: 10.1371/journal.pone.0075788 24116074 PMC3792128

[23] Constantinescu SN Girardot M Pecquet C. Mining for JAK-STAT mutations in cancer. Trends Biochem Sci (2008) 33(3):122–31. doi: 10.1016/j.tibs.2007.12.002 18291658

[24] Girardot M Pecquet C Boukour S Knoops L Ferrant A Vainchenker W. miR-28 is a thrombopoietin receptor targeting microRNA detected in a fraction of myeloproliferative neoplasm patient platelets. Blood (2010) 116(3):437–45. doi: 10.1182/blood-2008-06-165985 20445018

[25] Pecquet C Staerk J Chaligné R Goss V Lee KA Zhang X. Induction of myeloproliferative disorder and myelofibrosis by thrombopoietin receptor W515 mutants is mediated by cytosolic tyrosine 112 of the receptor. Blood (2010) 115(5):1037–48. doi: 10.1182/blood-2008-10-183558 19996410

[26] Dorritie KA McCubrey JA Johnson DE. STAT transcription factors in hematopoiesis and leukemogenesis: Opportunities for therapeutic intervention. Leukemia (2014) 28(2):248–57. doi: 10.1038/leu.2013.192 23797472

[27] Lu G Shi W Zheng H. Inhibition of STAT6/Anoctamin-1 activation suppresses proliferation and invasion of gastric cancer cells. Cancer Biother Radiopharm (2018) 33(1):3–7. doi: 10.1089/cbr.2017.2287 29466035

[28] Binnemars-Postma K Bansal R Storm G Prakash J. Targeting the Stat6 pathway in tumor-associated macrophages reduces tumor growth and metastatic niche formation in breast cancer. FASEB J (2018) 32(2):969–78. doi: 10.1096/fj.201700629R 29066614

[29] Tariq M Zhang JQ Liang GK He QJ Ding L Yang B. Gefitinib inhibits M2-like polarization of tumor-associated macrophages in Lewis lung cancer by targeting the STAT6 signaling pathway. Acta Pharmacol Sin (2017) 38(11):1501–11. doi: 10.1038/aps.2017.124 PMC5672074 29022575

[30] Wang N Tao L Zhong H Zhao S Yu Y Yu B. miR-135b inhibits tumour metastasis in prostate cancer by targeting STAT6. Oncol Lett (2016) 11(1):543–50. doi: 10.3892/ol.2015.3970 PMC4727074 26870245

[31] Li T Fan J Wang B Traugh N Chen Q Liu JS. TIMER: A web server for comprehensive analysis of tumor-infiltrating immune cells. Cancer Res (2017) 77(21):e108–10. doi: 10.1158/0008-5472.CAN-17-0307 PMC6042652 29092952

[32] Li B Severson E Pignon JC Zhao H Li T Novak J. Comprehensive aanalyses of tumor immunity: Implications for cancer immunotherapy. Genome Biol (2016) 17(1):174. doi: 10.1186/s13059-016-1028-7 27549193 PMC4993001

[33] Gao J Aksoy BA Dogrusoz U Dresdner G Gross B Sumer SO. Integrative analysis of complex cancer genomics and clinical profiles using the cBioPortal. Sci Signal (2013) 6(269):pl1. doi: 10.1126/scisignal.2004088 23550210 PMC4160307

[34] Liu CJ Hu FF Xia MX Han L Zhang Q Guo AY. GSCALite: a web server for gene set cancer analysis. Bioinformatics (2018) 34(21):3771–72. doi: 10.1093/bioinformatics/bty411 29790900

[35] Warde-Farley D Donaldson SL Comes O Zuberi K Badrawi R Chao P. The GeneMANIA prediction server: Biological network integration for gene prioritization and predicting gene function. Nucleic Acids Res (2010) 38(Web Server issue):W214–20. doi: 10.1093/nar/gkq537 PMC2896186 20576703

[36] Tang Z Li C Kang B Gao G Li C Zhang Z. GEPIA: a web server for cancer and normal gene expression profiling and interactive analyses. Nucleic Acids Res (2017) 45(W1):W98–W102. doi: 10.1093/nar/gkx247 28407145 PMC5570223

[37] Zhou Y Zhou B Pache L Chang M Khodabakhshi AH Tanaseichuk O. Metascape provides a biologist-oriented resource for the analysis of systems-level datasets. Nat Commun (2019) 10(1):1523. doi: 10.1038/s41467-019-09234-6 30944313 PMC6447622

[38] Miller AL Garcia PL Fehling SC Gamblin TL Vance RB Council LN. The BET inhibitor JQ1 augments the antitumor efficacy of gemcitabine in preclinical models of pancreatic cancer. Cancers (Basel) (2021) 13(14):3470. doi: 10.3390/cancers13143470 34298684 PMC8303731

[39] Fourniols T Maggio V Rafael D Colaco A Vidal EG Lopes A. Colorectal cancer inhibition by BET inhibitor JQ1 is MYC-independent and not improved by nanoencapsulation. Eur J Pharm Biopharm (2022) 171:39–49. doi: 10.1016/j.ejpb.2021.10.017 34998911

[40] Rudin CM Hann CL Garon EB Ribeiro de Oliveira M Bonomi PD Camidge DR. Phase II study of single-agent navitoclax (ABT-263) and biomarker correlates in patients with relapsed small cell lung cancer. Clin Cancer Res (2012) 18(11):3163–69. doi: 10.1158/1078-0432.CCR-11-3090 PMC3715059 22496272

[41] Joly F Fabbro M Follana P Lequesne J Medioni J Lesoin A. A phase II study of navitoclax (ABT-263) as single agent in women heavily pretreated for recurrent epithelial ovarian cancer: The MONAVI - GINECO study. Gynecol Oncol (2022) 165(1):30–9. doi: 10.1016/j.ygyno.2022.01.021 35123771

[42] Gotthardt D Putz EM Straka E Kudweis P Biaggio M Poli V. Loss of STAT3 in murine NK cells enhances NK cell-dependent tumor surveillance. Blood (2014) 124(15):2370–79. doi: 10.1182/blood-2014-03-564450 25185262

[43] Hossain DM Dos Santos C Zhang Q Kozlowska A Liu H Gao C. Leukemia cell-targeted STAT3 silencing and TLR9 triggering generate systemic antitumor immunity. Blood (2014) 123(1):15–25. doi: 10.1182/blood-2013-07-517987 24169824 PMC3879904

[44] Kujawski M Zhang C Herrmann A Reckamp K Scuto A Jensen M. Targeting STAT3 in adoptively transferred T cells promotes their *in vivo* expansion and antitumor effects. Cancer Res (2010) 70(23):9599–610. doi: 10.1158/0008-5472.CAN-10-1293 PMC3017475 21118964

[45] Siegel AM Heimall J Freeman AF Hsu AP Brittain E Brenchley JM. A critical role for STAT3 transcription factor signaling in the development and maintenance of human T cell memory. Immunity (2011) 35(5):806–18. doi: 10.1016/j.immuni.2011.09.016 PMC3228524 22118528

[46] Kaplan MH. STAT4: a critical regulator of inflammation *in vivo* . Immunol Res (2005) 31(3):231–42. doi: 10.1385/IR:31:3:231 15888914

[47] Bacon CM Petricoin EF 3rd Ortaldo JR Rees RC Larner AC Johnston JA. Interleukin 12 induces tyrosine phosphorylation and activation of STAT4 in human lymphocytes. Proc Natl Acad Sci U.S.A. (1995) 92(16):7307–311. doi: 10.1073/pnas.92.16.7307 PMC41328 7638186

[48] Hebenstreit D Wirnsberger G Horejs-Hoeck J Duschl A. Signaling mechanisms, interaction partners, and target genes of STAT6. Cytokine Growth Factor Rev (2006) 17(3):173–88. doi: 10.1016/j.cytogfr.2006.01.004 16540365

[49] Martinez FO Helming L Gordon S. Alternative activation of macrophages: an immunologic functional perspective. Annu Rev Immunol (2009) 27:451–83. doi: 10.1146/annurev.immunol.021908.132532 19105661

[50] Ansel KM Djuretic I Tanasa B Rao A. Regulation of Th2 differentiation and Il4 locus accessibility. Annu Rev Immunol (2006) 24:607–56. doi: 10.1146/annurev.immunol.23.021704.115821 16551261

[51] Yao Y Li W Kaplan MH Chang CH. Interleukin (IL)-4 inhibits IL-10 to promote IL-12 production by dendritic cells. J Exp Med (2005) 201(12):1899–903. doi: 10.1084/jem.20050324 PMC2212025 15967820

[52] Yoshihara K Shahmoradgoli M Martínez E Vegesna R Kim H Torres-Garcia W. Inferring tumour purity and stromal and immune cell admixture from expression data. Nat Commun (2013) 4:2612. doi: 10.1038/ncomms3612 24113773 PMC3826632

